# Supplementary material for: Interferon regulates neural stem cell function at all ages by orchestrating mTOR and cell cycle
Source: EMBO Mol Med. 2023 Jan 13;15(4):e16434. doi: 10.15252/emmm.202216434 (PMC10086582; doi:10.15252/emmm.202216434)
Supplement: Supplementary file 1 — Appendix [file EMMM-15-e16434-s003.pdf]

Appendix Data

Table of Contents

Appendix Figure S1: Ribo-Seq sample correlation quality control 1

Appendix Figure S2: Collection of western blot membranes for the biphasic response of mTOR 2

Appendix Figure S3: Polysome profiles of CRISPR-mediated TSC2 targetting of NSCs upon IFN-β treatment 3

Appendix supplementary methods: Modelling of neural stem cell dynamics in interferon knockout mice 4

Appendix Figure S4 5

Appendix Table S1 6

Appendix Table S2 7

Appendix Table S3 8

Appendix Figure S5 8

Appendix Figure S6 10

Appendix Table S4 11

Appendix Table S5 11

Appendix Table S6 12

Appendix Figure S7 13

Appendix Figure S8 14

Appendix Figure S9 15

Appendix Figure S1: Ribo-Seq sample correlation quality control

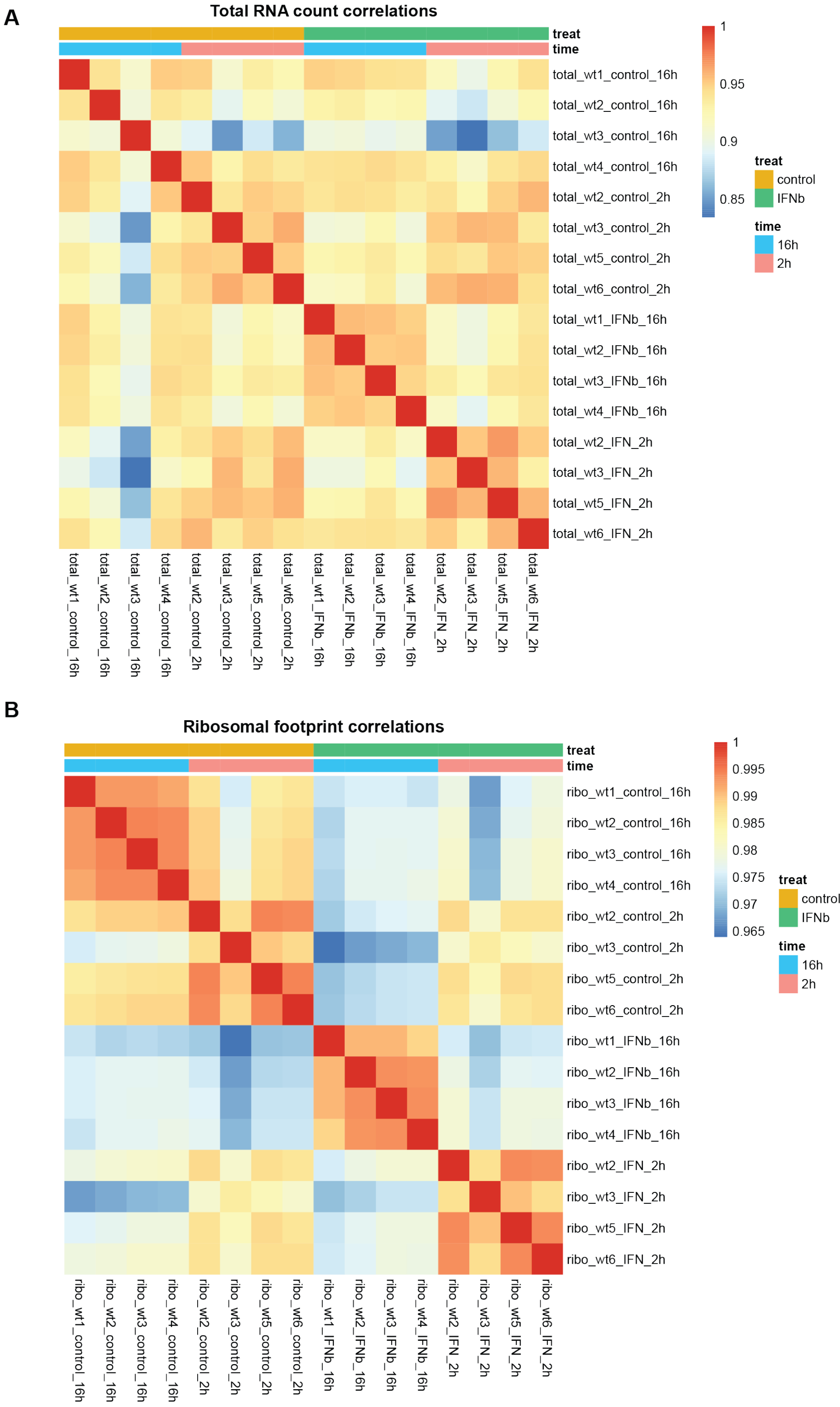

Appendix Figure S1. Ribo-Seq sample correlation quality control

A. Correlation matrix for ribosomal footprint libraries depicting pearson correlation coefficients for all samples. n = 4 biological replicates.

B. Correlation matrix for total RNA libraries depicting pearson correlation coefficients for all samples. n = 4 biological replicates.

Appendix Figure S2: Collection of western blot membranes for the biphasic response of mTOR

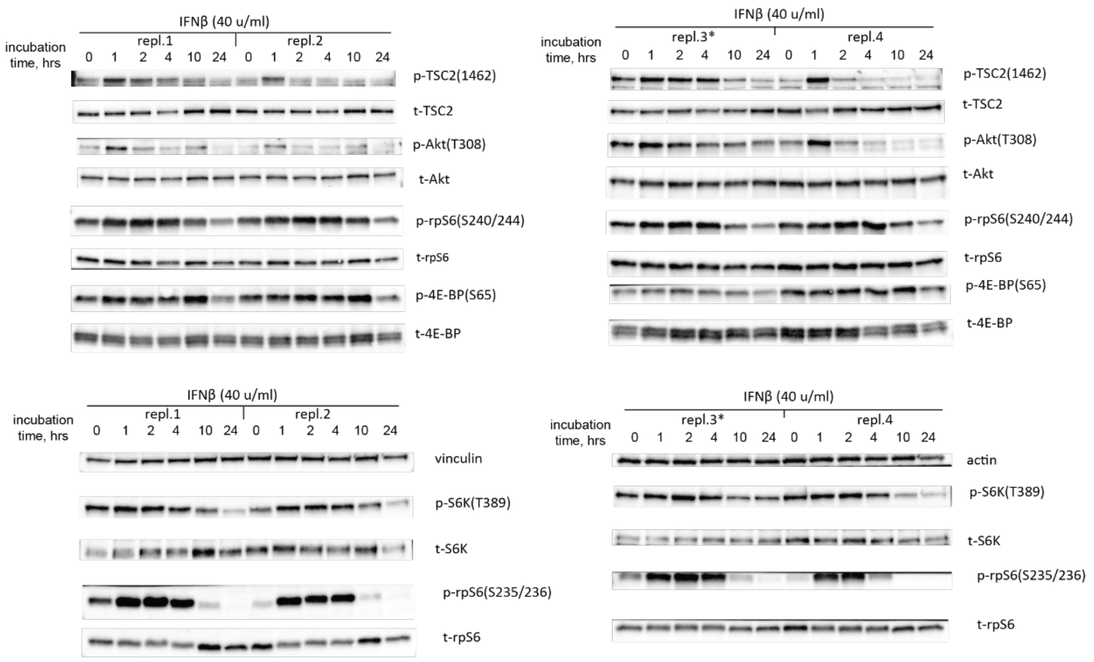

Appendix Figure S2. Collection of western blot membranes for assessing the biphasic response of mTOR caused by IFN-β treatment – Related to Fig 2I, 3A.

Collection of western blot membranes from 4 biological replicates of NSCs treated with IFN-β for the specified times. *Replicate 3 was used as representative blots for Figures 2I, 3A.

Appendix Figure S3: Polysome profiles of CRISPR-mediated TSC2 targetting of NSCs upon IFN-β treatment

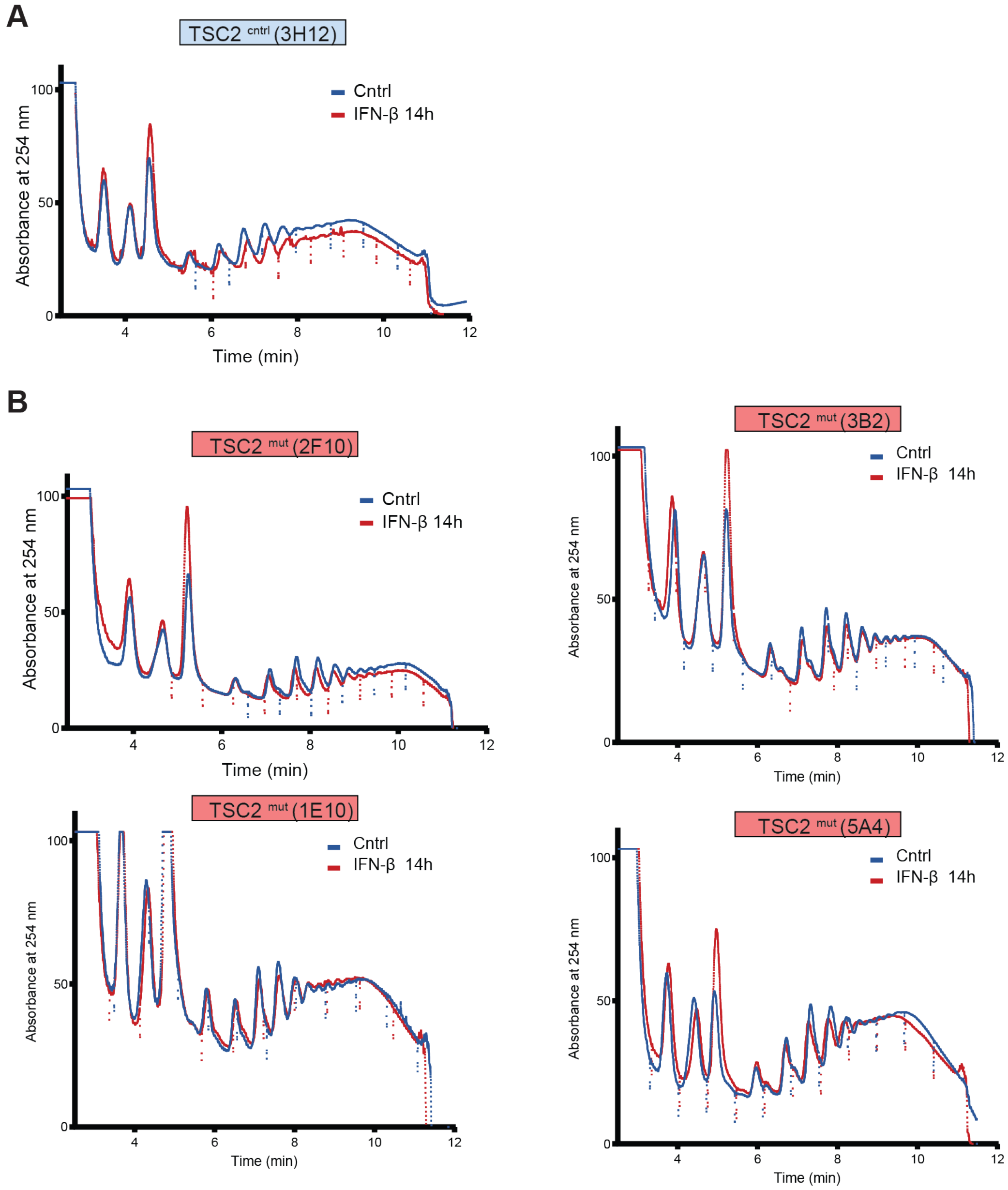

Appendix Figure S3. Polysome profiles of CRISPR-mediated TSC2 targetting of NSCs upon IFN-β treatment – Related to Fig EV4

A. Polysome profiles of CRISPR-mediated TSC2^{cntrl} NSC clone treated with IFN-β (red) as compared to control (blue).

B. Polysome profiles of CRISPR-mediated TSC2^{mut} NSC clones treated with IFN-β (red) as compared to control (blue).

Appendix supplementary methods: Modelling of neural stem cell dynamics in interferon knockout mice

Modelling of neural stem cell dynamics in interferon knockout mice

1 Model

The work is based on the models which were previously established for neurogenesis of the hippocampus [1–3] and recently extended and quantified for the ventricular sub-ventricular zone [4, 5]. We adapt the model to describe the NSC dynamics in the ventricular-subventricular zone (V-SVZ) of Interferon α and γ receptor knockout (IFNAGR KO) mice.

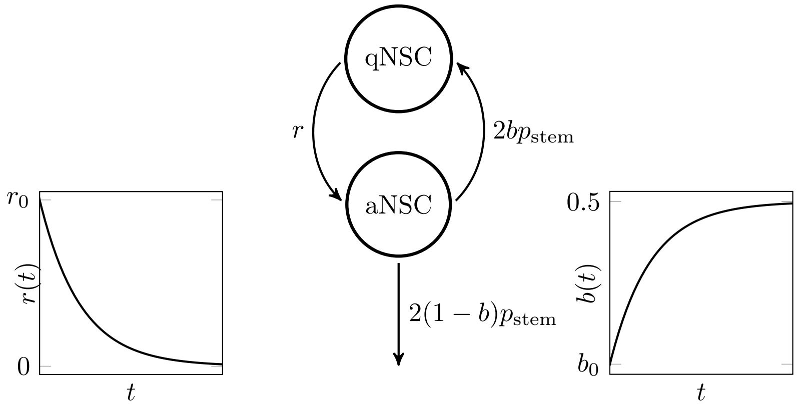

Appendix Figure S4: Graphical representation of the model and plots for parameter functions. qNSC activate at rate r, at which point they progress through cell cycle at rate p_{stem}, to either produce two qNSC at probability b, or at probability $1-b$ undergo differentiation.

The model consists of a system of ordinary differential equations

$$\frac{d}{dt}\text{qNSC}(t) = -r \cdot \text{qNSC}(t) + 2 \cdot b \cdot p_{\text{stem}} \cdot \text{aNSC}(t) \quad (1)$$

$$\frac{d}{dt}\text{aNSC}(t) = r \cdot \text{qNSC}(t) - p_{\text{stem}} \cdot \text{aNSC}(t) \quad (2)$$

where qNSC and aNSC describe the number of quiescent (non-cycling) and active (cycling) stem cells. Quiescent stem cells (qNSC) enter cell cycle at rate r, becoming active stem cells (aNSC). Parameter p_{stem} describes the division rate and b the fraction of self-renewal, reflecting the probability that a progeny cell stays a stem cell (qNSC) (Appendix Figure S4). Consequently, progenitor cells arise at the probability $1-b$.

In line with previous models, we assume a constant cell division rate, based on observed cell cycle lengths from Ponti et al. [6]:

$$p_{\text{stem}} = \log(2) \cdot 24 \cdot 17.5^{-1} \approx 0.95 \text{ days}^{-1}.$$

and introduce time-dependent parameters (plots in Appendix Figure S4),

$$r(t) = r_0 \cdot e^{-t \cdot \beta_r} \tag{3}$$

$$b(t) = \frac{1}{2}\left(1 + e^{-t \cdot \beta_b} \left(2 \cdot b_0 - 1\right)\right). \tag{4}$$

In the remainder of this paper, models with the time-dependent r are called the quiescence models, while the models with time-dependent b are referred to as the self-renewal models. The models with constant r and b are called no ageing models, as they assume that cell properties do not change during aging (Appendix Table S1).

Appendix Table S1: Model variants with fully time-dependent parameters fitted. Free parameters list which parameters were estimated in model fitting. NSC(0) is the sum of both NSC compartments, at time point zero.

Name	Description	Free parameters
No aging	No time-dependence	NSC(0), r, b
Quiescence	Decreasing activation rate	NSC(0), β_r, r_0, b
Self-renewal	Increasing self-renewal probability	NSC(0), r, β_b, b_0
Self-renewal and Quiescence	Increasing self-renewal probability and decreasing activation rate	NSC(0), β_r, r_0 β_b, b_0

ODE initial values were picked from the steady-state ratio of the system with constant parameters. The steady state ratio can be found by solving

$$\frac{d}{dt}\frac{\text{qNSC}}{\text{aNSC}} = 0$$

using (1) and (2), which reveals that

$$\frac{\text{qNSC}}{\text{aNSC}} = \frac{p_{\text{stem}} - r}{2r} + \sqrt{\left(\frac{p_{\text{stem}} - r}{2r}\right)^2 + 2\frac{b p_{\text{stem}}}{r}}. \tag{5}$$

This gives the full model the free parameters NSC_0, r_0, b_0, β_r, β_b (See Appendix Table S1 for exact parameters in each model).

2 IFNAGR KO parameter estimates

2.1 Data

We obtained data from two separate experiments. In the first experiment, fluorescence activated cell sorting (FACS) was used to quantify the total number of

neural stem cells (NSC) in the ventricular sub-ventricular zone. FACS quantifications for wildtype mice were taken from Kalamakis et al. Some FACS quantifications (Appendix Table S2) were acquired in pools of mice instead of individual mice, here referred to as 'quantifications'. IFNAGR KO NSC population estimates were obtained in eight quantifications at two months old, three quantifications at six month old and three quantifications at 22 months old (Appendix Table S2). In the second experiment, immunohistochemistry (IHC) was used to quantify the fraction of cycling (i.e. active) stem cells. This data is from Kalamakis et al. [4, Fig.5G]. By quantifying $Sox2^{+}Dcx^{-}BrdU^{+}Mki67^{+}$ as a fraction of $Sox2^{+}Dcx^{-}BrdU^{+}$ cells in a pulse-chase labelling regime this dataset avoids labelling cycling pro-genitors. Due to this, the fraction of cycling stem cells can now be computed as $\mathrm{aNSC}(t)/\mathrm{NSC}(t)$ from model outputs.

Appendix Table S2: Data used. Age is given in months. Numbers marked with † indicate that four of these quantifications were actually pools of two mice and four were individual quantifications. ‡ Indicates that all of these quantifications were pools of three mice.

		Quantifications	
Genotype	Age	FACS	IHC
IFNAGR KO	2	8†	3
	6	3	—
	22	3	4
Wildtype	2	8†	3
	7	3	—
	22	3‡	3

2.2 Parameter estimation

Parameter estimates were obtained using multi-start weighted least-squares optimisation. Optimisation starting values were sampled from optimised Latin Hypercubes using `LatinHypercubeSampling.jl` and scaled to optimisation bounds (Appendix Table S3). Initial value problems were created on the previously described steady-state ratios and solved using `DifferentialEquations.jl`'s solvers. Objective functions were built based on weighted least-squares. Weights used were the inverse of the standard deviation for each age and genotype combination. Op-timisation of objective functions was then performed using box-constrained op-timisers from the `Optim.jl` library. The minimum optimum for each model and genotype combination was chosen as the best optimum. For model comparison, the low-sample size adjusted Akaike Information Criterion (AIC_c) was calcu-lated for each model and genotype combination. The Akaike Information Cri-terion is an information-theoretic approach for model comparison that penalises extra parameters and rewards lower residuals. Lower AIC_c values correspond to "better" models.

2.3 Time-dependent models

Wildtype data was best explained by models with increasing quiescence or increasing quiescence and self-renewal (Appendix Table S4). Models with only increasing self-

Appendix Table S3: Allowed parameter ranges for box-bounded optimisation and start-ing values.

Parameter	Lower	Upper
NSC(0)	0.0	2500.0
r_0	0.0	1.0
b_0	0.0	0.5
β_b	0.0	0.1
β_r	0.0	0.1

renewal failed to account for the variation in active stem cell fractions. While including self-renewal in a quiescence model provides better residuals, the change in AIC_c is not large. Previously, this, along with the minute change in self-renewal probability, was considered insufficient evidence to assume that both are taking place. These parameter estimates (Appendix Table S5) nicely recapitulate the results from our previous publication [4].

Data for IFNAGR KO mice was not explainable by time-dependent decreases in activation ("Quiescence" model). Since wildtype mice show this decrease and have IFNAGR, this indicates that IFNAGR do indeed play a role in age-related activation changes. Should these changes in activation be the only mechanism at play, we would expect IFNAGR KO to have no more time-dependent effects. However, as there is a population of stem cells remaining in old IFNAGR KO mice, models where no parameters are changing in time ("No ageing" model) are not able to explain this data. To explain this data where no age-dependent activation changes can take place, yet a population of stem cells remains in age, a time-dependent self-renewal probability is necessary. Indeed, models with such a time-dependent increase in self-renewal are the best-performing models for this dataset when ranked using residuals or AIC_c (Appendix Table S4).

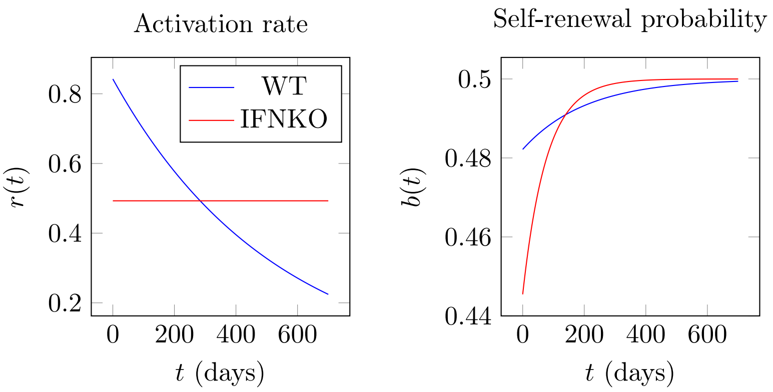

Appendix Figure S5: Comparison of parameter estimates for combined quiescence and self-renewal models. The left panel shows the rate of activation, r, the right panel the probability of self-renewal, b.

While it was previously argued that this small change in self-renewal probability is not meaningful due to the much larger changes in activation rate, changes on this scale are in fact meaningful for populations and sufficient to maintain

them (Appendix Figures S5-6). With time-dependent self-renewal improving fits in both wildtype and interferon deficient mice as well as it's ability to maintain stem cell populations, it seems likely that time-dependent (or population-dependent) self-renewal is a second layer of stem cell maintenance that exists in wildtype mice as well and becomes obvious once ageing-induced quiescence is removed, as is the case in IFNAGR KO mice.

2.4 Population-dependent self-renewal

Stem cell populations approach the plateau of approximately 150 cells in wild-type mice faster than they do in knockout mice (Appendix Figure S6), which coincides with a faster increase in self-renewal probability in models that allow for changing self-renewal. This hints at a possible population-dependence of the self-renewal term. To address this issue, we additionally fit models where self-renewal was expressed in terms of total stem cells. To do this, we employed a Hill function:

$$b(\mathrm{NSC}) = 1 - \frac{1}{1 + \left(\frac{k_b}{\mathrm{NSC}}\right)^{n_b}} \tag{6}$$

This parameterisation of the Hill function exposes the population size at $b = 0.5$ as k_b. As this system has a steady state at $b = 0.5$, k_b is where the population will plateau.

To address if IFNAGR have a possible effect on b,we fit both data sets at the same time. To do this, we created two different models: In the first all parameters of b are shared. This models self-renewal not being affected by interferon (IFN-independent self-renewal). In the second we allowed n_b to vary with genotype. As the plateau of both populations was the same, we did not allow k_b to change with genotype. This simulates the case that interferon has an effect on self-renewal (IFN-dependent self-renewal).

Both models were capable of explaining the observed data. The IFN-dependent model provides a small benefit to the fit at the cost of one extra parameter. Because of this, at this time it is not clear if IFNAGR additionally affects self-renewal and further experiments are required to determine this.

3 Conditional knockout simulation

To investigate the age-dependent effects of interferon knockouts, we built models that simulate an Interferon receptor knockout induced at a specific age. These models work the same as our wildtype models with population-dependent self-renewal up to some timepoint of intervention t_1. At this timepoint, the parameter functions are switched to the functions quantified on the IFNAGR KO data and the model continues running with these. Due to the uncertain nature of the effects of IFNAGR KO on self-renewal we simulated this model with both the shared b(NSC) and IFN-dependent b(NSC) individually.

We considered two metrics of proper niche functioning: The size of the population as well as the production of progenitors from stem cells. This rate of progenitor production follows from (1) as

$$\mathrm{aNSC}(t) \cdot (1 - b) \cdot 2 \cdot p_s. \tag{7}$$

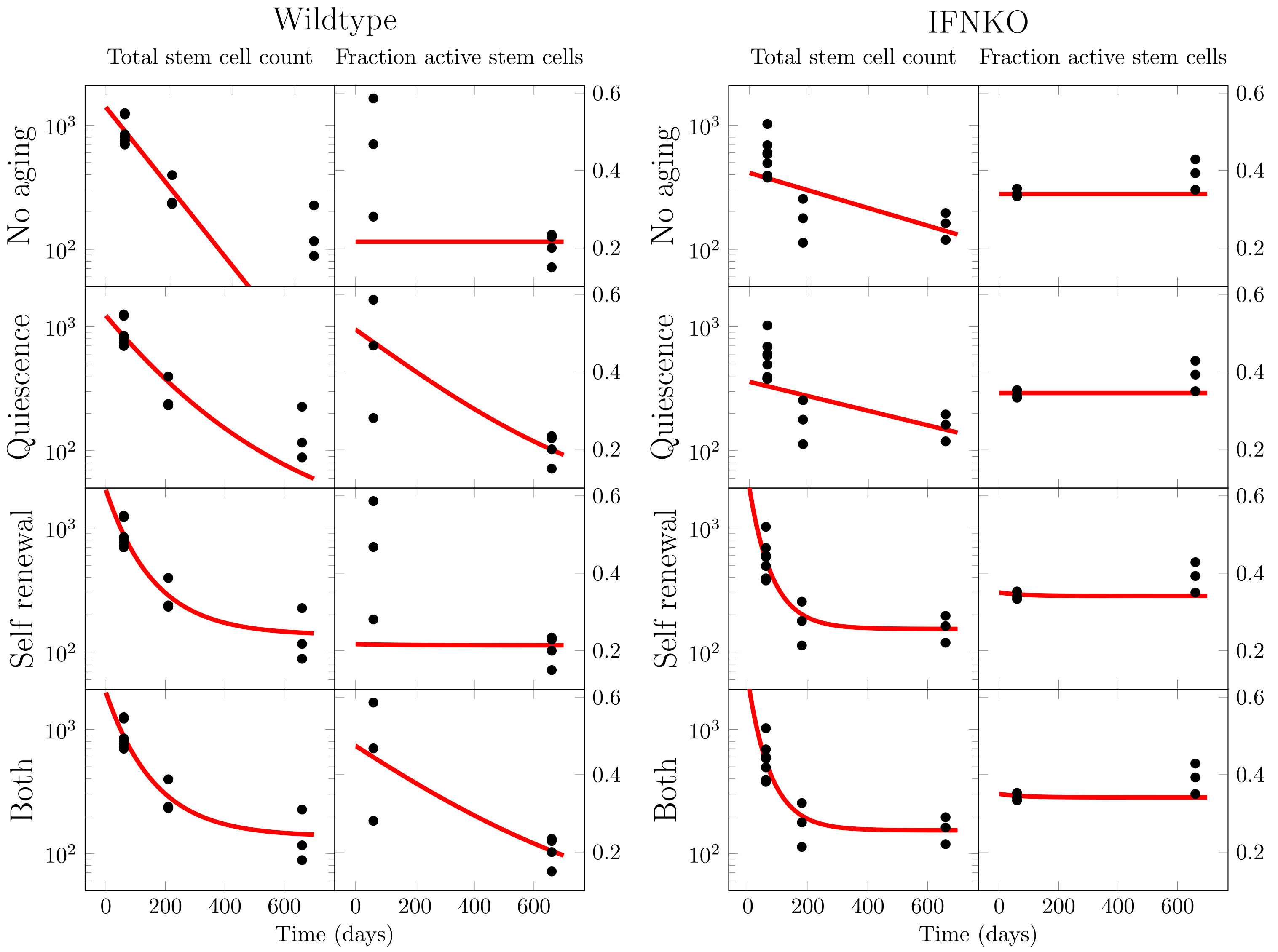

Appendix Figure S6: Models fitted to wildtype (left column) and interferon knockout (right column) data. Panels on the left show total amounts of stem cells, qNSC + aNSC. Panels on the right show the fraction of cycling cells among these, aNSC/NSC. Red lines represent model solutions and black dots are measurements. Each row is a separate model, "Both" is the model with both time-dependent self-renewal and quiescence.

Appendix Table S4: Model fit comparison. ΔAIC_c is the change in AIC_c provided by this model compared to the best model (lowest AIC_c) for the given genotype. r_w are the weighted residuals that were optimised for. Models are sorted by ΔAIC_c, putting the top-performing models at the top.

	Model	r_w	ΔAIC_c
IFNKO	Self renewal	20.28	0.0
	Self renewal and Quiescence	20.28	1.8
	No ageing	36.66	10.25
	Quiescence	34.95	10.89
Wildtype	Self renewal and Quiescence	16.0	0.0
	Quiescence	21.61	4.55
	Self renewal	22.94	5.81
	No ageing	33.1	11.93

Appendix Table S5: Obtained parameter estimates for wildtype and interferon knock-out data. Values at '—' are not present and can be read as 0.

	parameter	no ageing	quiescence	self-renewal	both
IFNAGR KO	NSC_0	412.9	359.0	2500.0	2500.0
	r_0	0.4878	0.5006	0.4928	0.4928
	β_r	—	$2.56 \cdot 10^{-10}$	—	$1.94 \cdot 10^{-12}$
	b_0	0.4975	0.4987	0.4455	0.4455
	β_b	—	—	0.01288	0.01288
Wildtype	NSC_0	1397.0	1228.0	2031.0	1986.0
	r_0	0.2598	0.9786	0.2587	0.8426
	β_r	—	0.00216	—	0.001891
	b_0	0.4821	0.4932	0.4596	0.4822
	β_b	—	—	0.006139	0.004862

Appendix Table S6: Residuals for models with population dependent self-renewal. The number of free parameters is denoted k, the residuals r_w and the difference in low-sample size corrected Akaike Information Criterion ΔAIC_c.

Self-renewal	k	r_w	ΔAIC_c
IFN-independent	8	39.0	
IFN-dependent	9	36.6	

To assess the life-long effects o f t his c onditional k nockout w e a lso computed the integral of this progenitor production from 0 to 700 days, here called "life-long progenitor production".

3.1 IFN-dependent self-renewal

Conditional knockout simulations with IFN-dependent self-renewal showed a decrease of stem cell populations upon intervention for all intervention ages. This difference w as m o s t p r o nounced i n y oung a g e s, w h e re t h e p o p ulation was down to 60% of wildtype in the worst case. In old ages, this effect was less pronounced, with mice retaining most of their stem cells (Appendix Figure S7a-b). In young ages, progenitor production was also seriously affected. This was down to 50%of the wildtype in the worst cases, but eventually recovered to yield a higher progenitor production than in wildtype for old ages, up to 175% of the wildtype production (Appendix Figure S7c-d). At ages beyond approximately 100 days, the total num-ber of cells produced was higher than in wildtype, peaking out around 300 days intervention age. Overall, this shows that IFNAGR KO could increase the pro-ductivity of the niche at the cost of a few cells, when done in sufficiently old ages.

3.2 IFN-independent self-renewal

Simulations with IFN-independent self-renewal show the same trend of increased progenitor production in older ages, with some decrease in stem cell numbers in old ages as the IFN-dependent self-renewal models (Appendix Figure S8a). Contrary to those however, there is no loss of stem cells in young interventions. Instead, stem cell populations increase in size for interventions in young ages (Appendix Figure S8b). Further, there is no strong loss of progenitor production in early ages either (Appendix Figure S8c-d). Thus, should IFNAGR also have an effect on self-renewal, progenitor production would still be increased in age, with no obvious adverse effects in young ages (Appendix Figure S9).

References

[1] Frederik Ziebell, Ana Martin-Villalba and Anna Marciniak-Czochra. "Mathematical modelling of adult hippocampal neurogenesis: effects of altered stem cell dynamics on cell counts and bromodeoxyuridine-labelled cells". In: *Journal of The Royal Society Interface* 11.94 (6th May 2014), p. 20140144. DOI: 10.1098/rsif.2014.0144. URL: https://royalsocietypublishing.org/doi/full/10.1098/rsif.2014.0144 (visited on 20/09/2019).

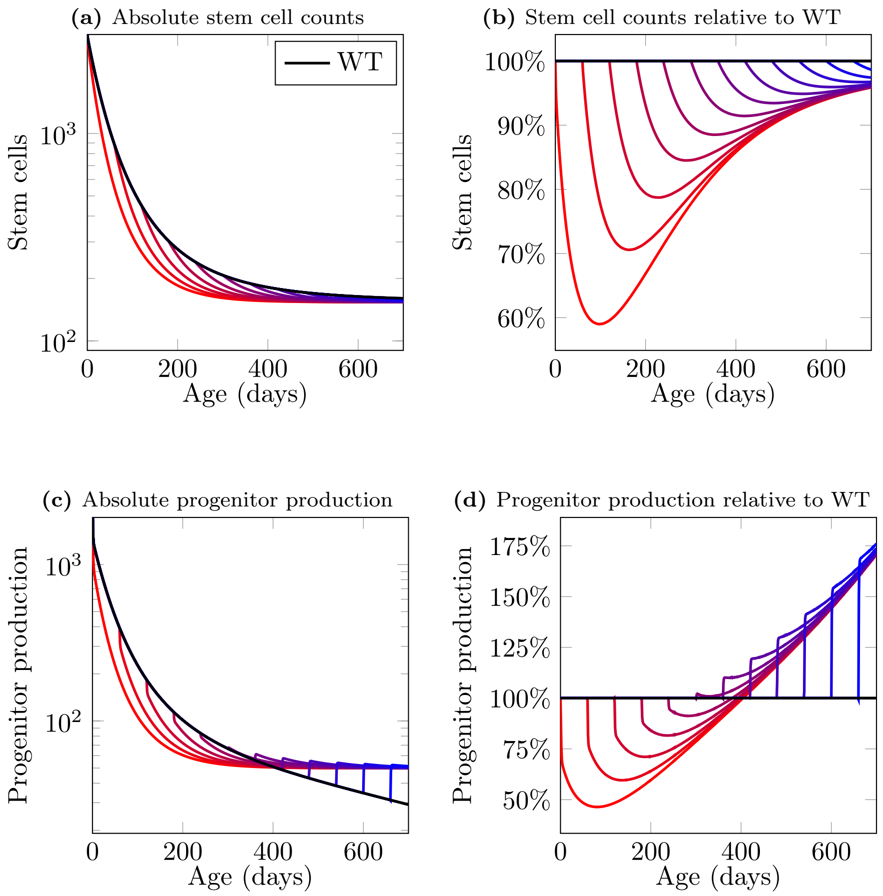

Appendix Figure S7: IFN-dependent conditional knockout simulations. Stem cell counts are the total stem cells for the wildtype (black) and simulations at varying timepoints (coloured from red to blue). Progenitor production is the rate of progenitors produced from stem cells. Relative values are expressed relative to the WT simulation.

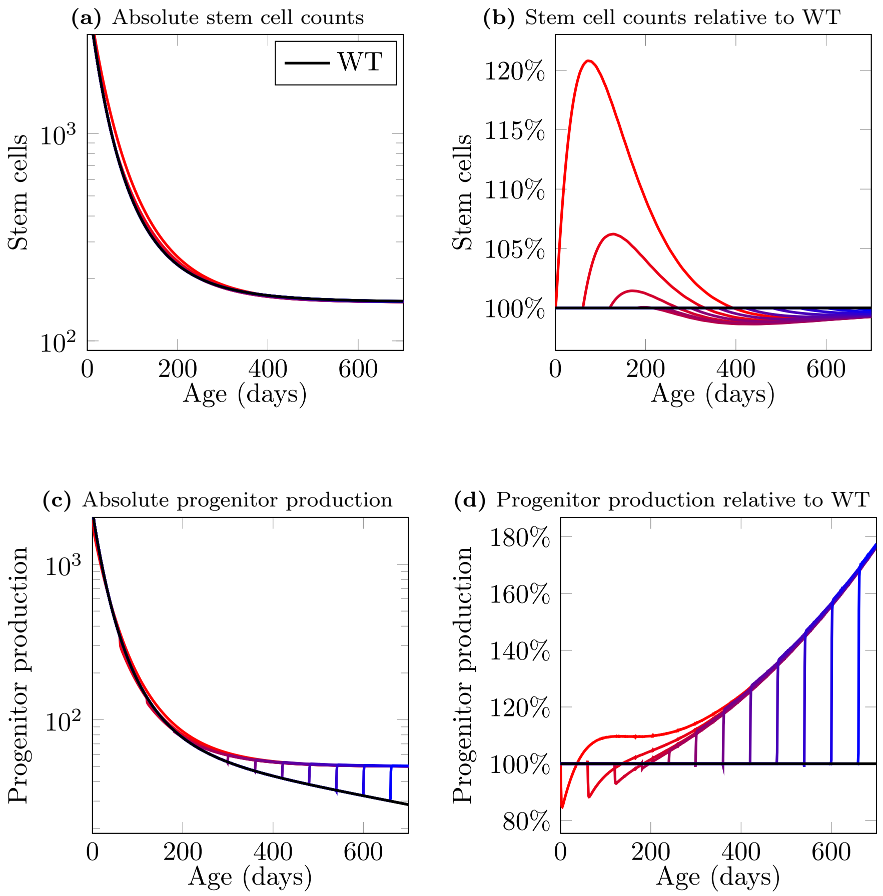

Appendix Figure S8: IFN-independent conditional knockout simulations. Stem cell counts are the total stem cells for the wildtype (black) and simulations at varying timepoints (coloured from red to blue). Progenitor production is the rate of progenitors produced from stem cells. Relative values are expressed relative to the WT simulation.

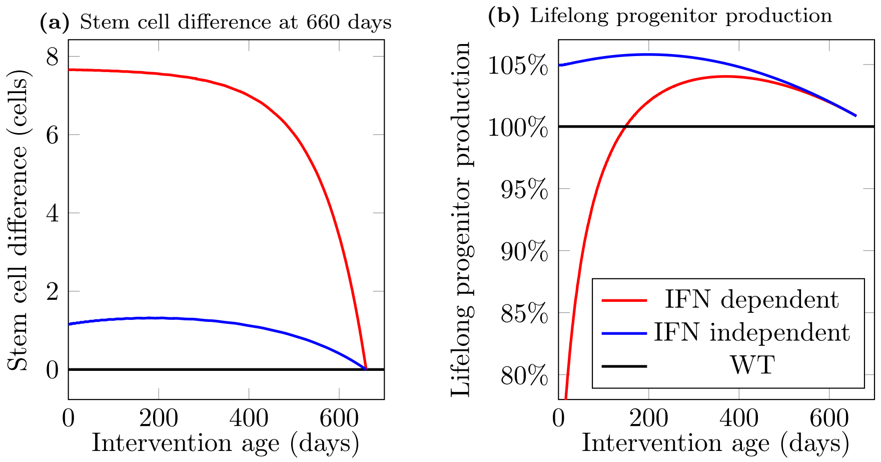

Appendix Figure S9: Stem cell differences at old age (660 days) for varying intervention ages and life-long progenitor production as percentage of wildtype. Both a scenario with interferon dependent (red) and interferon independent (blue) self-renewal are shown compared to the wildtype (black).

[2] Frederik Ziebell et al. "Revealing age-related changes of adult hippocampal neurogenesis using mathematical models". In: *Development* 145.1 (Jan. 2018). dev153544. ISSN: 0950-1991. DOI: `10.1242/dev.153544`. eprint: `https://journals.biologists.com/dev/article-pdf/145/1/dev153544/1852642/dev153544.pdf`. URL: `https://doi.org/10.1242/dev.153544`.

[3] L. Harris et al. "Coordinated changes in cellular behavior ensure the lifelong maintenance of the hippocampal stem cell population". In: *Cell Stem Cell* 28 (2020), 863–876. e6.

[4] Georgios Kalamakis et al. "Quiescence Modulates Stem Cell Maintenance and Regenerative Capacity in the Aging Brain". In: *Cell* 176.6 (Mar. 2019), 1407–1419.e14. ISSN: 00928674. DOI: `10.1016/j.cell.2019.01.040`. URL: `https://linkinghub.elsevier.com/retrieve/pii/S0092867419301035` (visited on 12/07/2019).

[5] Lukas PM. Kremer et al. "In vivo high-throughput screening of novel adeno-associated viral capsids identifies variants for transduction of adult neural stem cells within the subventricular zone". In: *Molecular Therapy - Methods & Clinical Development* (2021). ISSN: 2329-0501. DOI: `https://doi.org/10.1016/j.omtm.2021.07.001`. URL: `https://www.sciencedirect.com/science/article/pii/S2329050121001182`.

[6] Giovanna Ponti et al. "Cell cycle and lineage progression of neural progenitors in the ventricular-subventricular zones of adult mice". In: *Proceedings of the National Academy of Sciences* 110.11 (2013), E1045–E1054. ISSN: 0027-8424. DOI: `10.1073/pnas.1219563110`. eprint: `https://www.pnas.org/content/110/11/E1045.full.pdf`. URL: `https://www.pnas.org/content/110/11/E1045`.
