# Supplementary material for: Interferon regulates neural stem cell function at all ages by orchestrating mTOR and cell cycle
Source: EMBO Mol Med. 2023 Jan 13;15(4):e16434. doi: 10.15252/emmm.202216434 (PMC10086582; doi:10.15252/emmm.202216434)
Supplement: Supplementary file 2 — Expanded View Figures PDF [file EMMM-15-e16434-s006.pdf]

Expanded View Figures

Figure EV1. Inference of IFN signatures in the vSVZ niche.

A Experimental layout for scRNA-Seq in young and old mice lacking interferon receptors. All mice represent TiCY (Tlx reporter, see Materials and Methods and Fig EV1) that are either IFNAGR WT or KO. vSVZ (Ventricular Subventricular Zone), RMS (Rostral Migratory Stream), OB (Olfactory Bulb), TAM (Tamoxifen).

B Relative gene expression of relevant markers for cell types along pseudotime. Black lines denote cuts between cell types.

C Relative expression of type-I IFN receptors in young and old wildtype cells over pseudotime coloured by lineage cell types.

D Scores computed for the Hallmark Interferon Alpha Response signature displayed in the UMAP embedding for young cells (with colours clipped to the range seen in the lineage cells) and averaged for the cell types in our analysis at varying ages in in $IFNAGR^{WT}$ and $IFNAGR^{KO}$ cells. $n = 2$ biological replicates per age and genotype.

E Scores computed for the Hallmark Inflammatory Response signature displayed in the UMAP embedding for young cells (with colours clipped to the range seen in the lineage cells) and averaged for the cell types in our analysis at varying ages in $IFNAGR^{WT}$ and $IFNAGR^{KO}$ cells. $n = 2$ biological replicates per age and genotype.

© 2023 The Authors

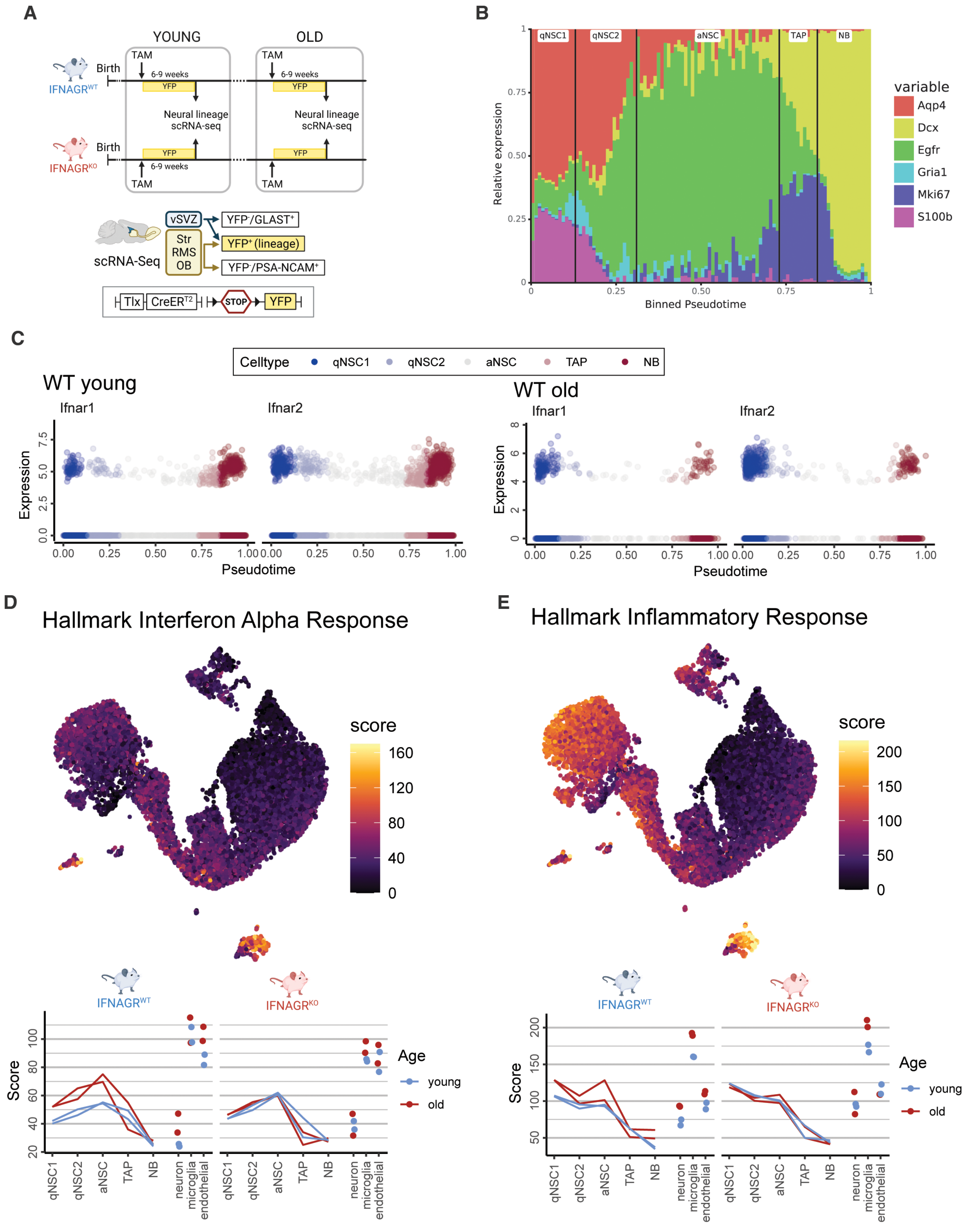

Figure EV1.

© 2023 The Authors

Figure EV2. Cycleflow and OPP analysis: IFN-β induce cell cycle exit and a biphasic control of protein translation in NSCs.

A Gating strategy for quantification of cell cycle states for Cycleflow.
B EdU and IFN-β exposure experimental scheme for Cycleflow.
C Cell cycle properties inferred from Cycleflow in IFN-β-treated NSCs. $n = 3$ biological replicates.
D Representative images of the OPP (O-propargyl-puromycin) incorporation assay in WT NSCs treated with IFN-β at 2 and 16 h. N2 supplement or Cycloheximide (CHX) where added as controls (see Materials and Methods). Bar scale represents 50 μm.
E OPP (O-propargyl-puromycin) incorporation assay quantification in WT NSCs treated with IFN-β at 2 and 16 h. $n = 5$ biological replicates. One-way ANOVA with Dunnett's multiple comparison test was computed (*P*-values specified) using vehicle-treated NSCs as control group.

© 2023 The Authors

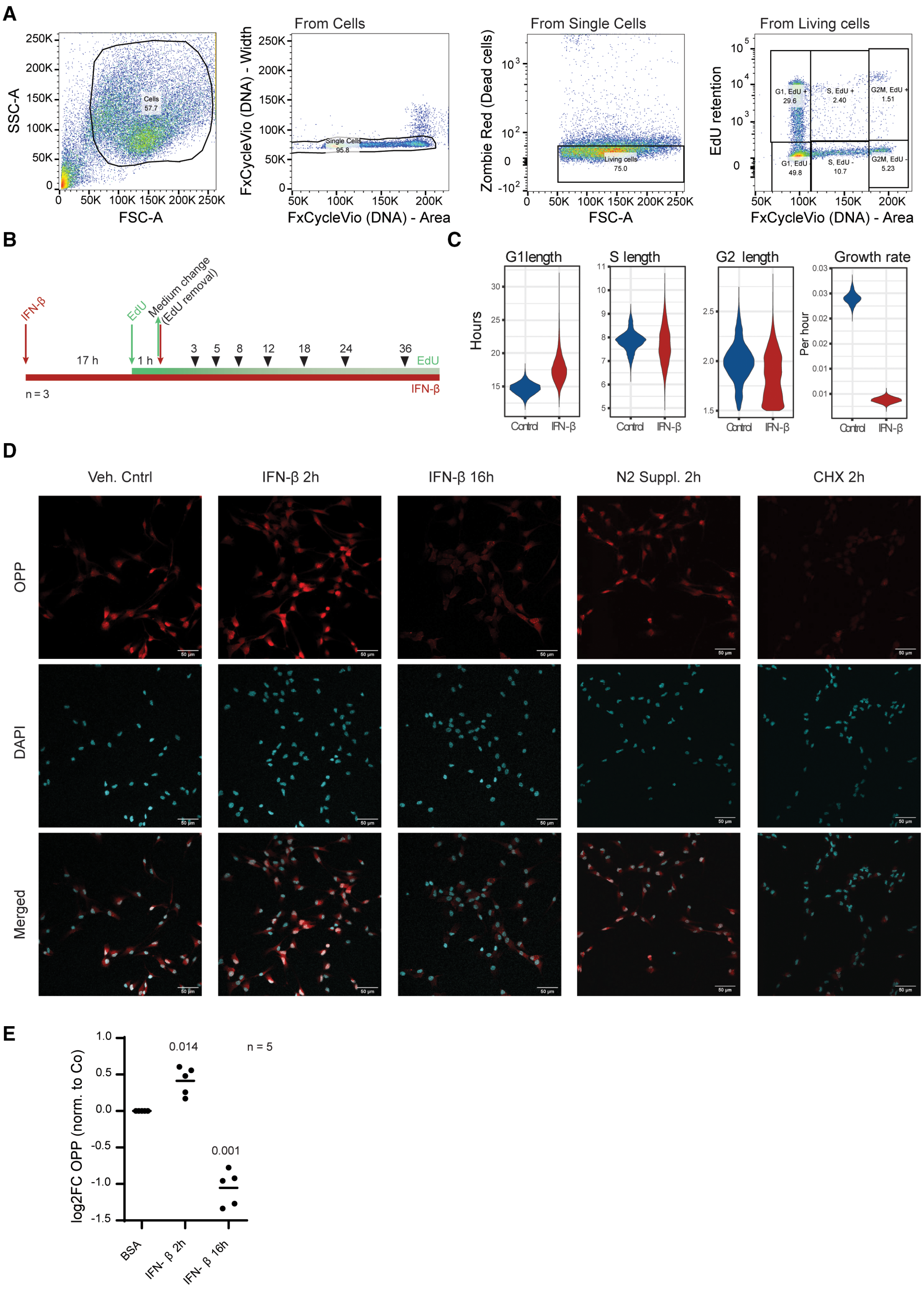

Figure EV2.

 © 2023 The Authors

Figure EV3. IFN-β induce a biphasic control of mRNA translation in NSCs.

A, B Results of Ribo-Seq depicting translation efficiency as the interaction of log2 fold changes (LFC) between footprints (ribosome protected reads) and total RNA at 2 and 14 h IFN-β treatment. FDR < 10%, LR-Test. Genes with a *P*-value < 0.1 after FDR correction are highlighted. The associated GO terms are depicted. $n = 3$ biological replicates.

C Polysome profiling (RT-qPCR) of actin beta and of the interferon stimulated genes (ISGs) Ifit3 and Irf9 upon 2 and 14 h IFN-β treatment. Hyphens represent mean of biological replicates. Arrows indicate the 40S, 60S and 80S subunits of the ribosome. Two-way ANOVA with Šídák's multiple comparison test was computed. Two hours Ifit3 fraction 4 ***($P < 0.0001$), fraction 10 **($P = 0.0024$); 2 h Irf9 fractions 5, 6, 10 ***($P < 0.0001$), fraction 9 **($P = 0.0091$). Fourteen hours Actb fractions 9, 10 ***($P < 0.0001$), fraction 11 ***($P = 0.0002$); 14 h Ifit3 fraction 10 ***($P < 0.0001$), fraction 11 ***($P = 0.0003$); 14 h Irf9 fraction 1 *($P = 0.0093$), fractions 2, 3, 5 ***($P < 0.0001$), fraction 4 *($P = 0.0104$), fraction 6 *($P = 0.0263$), fraction 10 **($P = 0.0092$). $n = 3$–4 biological replicates. Outliers of fraction 1 from Ifit3 were excluded from the statistical analysis.

D Polysome profiling (RT-qPCR) of *Rps17* and *Rpl34* upon 2 and 14 h IFN-β treatment. Hyphens represent mean of biological replicates. Arrows indicate the 40S, 60S and 80S subunits of the ribosome. Two-way ANOVA with Šídák's multiple comparison test was computed. Two hours Rpl34 fraction 7 *($P = 0.037$). Fourteen hours Rps17 fraction 4 ***($P = 0.0002$), fraction 5 **($P = 0.003$), fraction 7 ***($P < 0.0001$), fraction 8 **($P = 0.0018$); 14 h Rpl34 fractions 4, 7 ***($P < 0.0001$), fraction 5 ($P = 0.0047$), fraction 8 **($P = 0.0042$). $n = 3$–4 biological replicates.

E Representative WB image and quantification (log2FC) of LARP1 from IFN-β-treated NSCs normalised to control ($t = 0$ h). Bars represent the mean value. $n = 4$ biological replicates.

F Representative WB image and quantification (log2FC) of p-Akt^{Ser473} from IFN-β-treated NSCs normalised to control ($t = 0$ h). Bars represent the mean value. One sample *t*-test was computed, no significant results. $n = 3$ biological replicates.

Data information: $P \equiv P$-value.

© 2023 The Authors

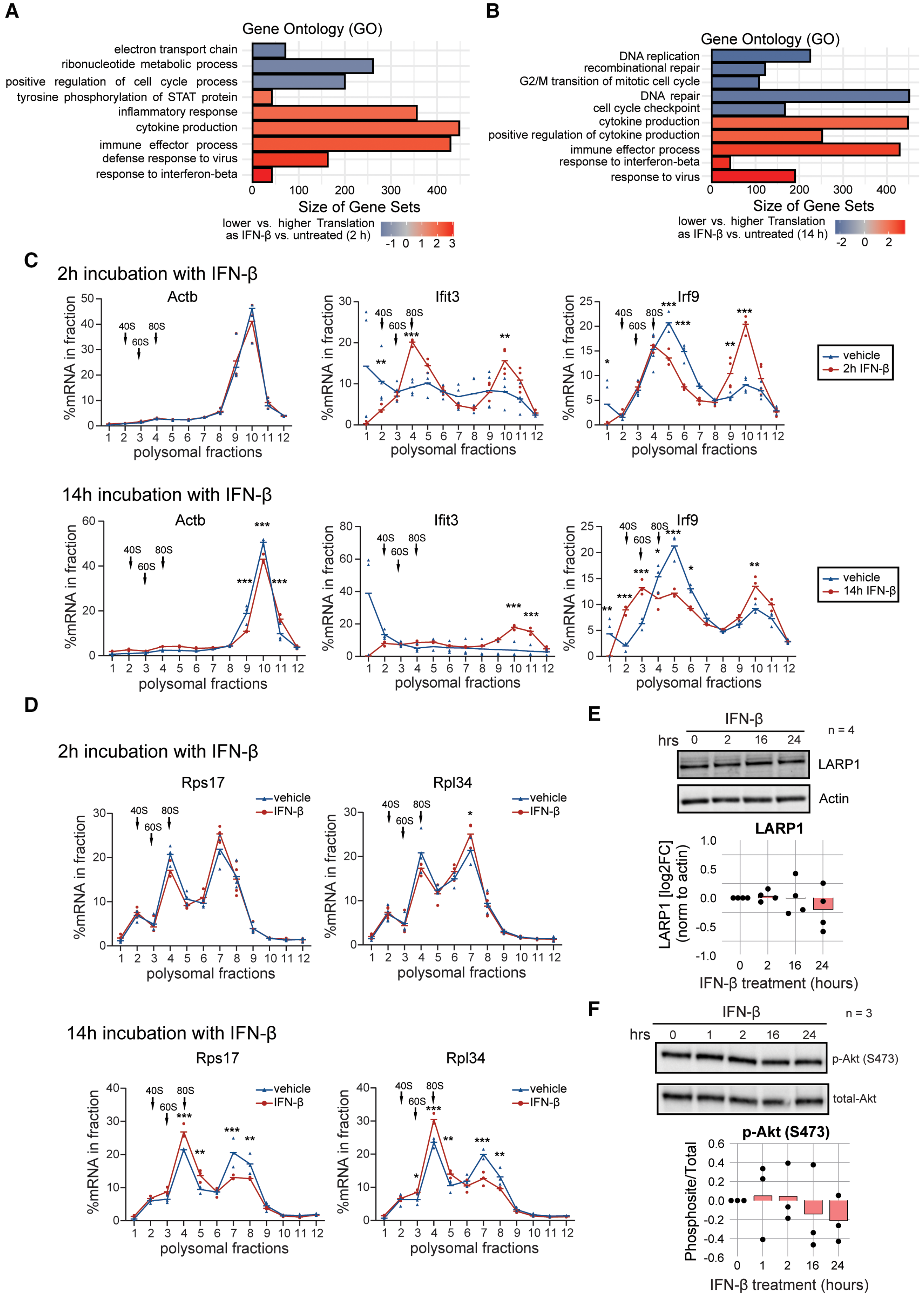

Figure EV3.

 © 2023 The Authors

Figure EV4. The biphasic control of mTORC1 by IFN-β relies on TSC2, PKR and eIF2α.

A WB images and quantifications of TSC2 relative to actin beta and normalised to $TSC2^{WT}$ NSCs in different CRISPR-mutated ($TSC2^{mut}$) or CRSPR-non-mutated ($TSC2^{cntrl}$) NSC clones.

B Representative WB images and quantifications (log2FC) of p-$p70S6K^{Thr389}$ in $TSC2^{WT}$, $TSC2^{cntrl}$ & $TSC2^{mut}$ NSCs treated with IFN-β and normalised to vehicle ($t = 0$ h) $TSC2^{WT}$. Bars represent the mean value. $n = 2$–4 clonal replicates.

C OPP (O-propargyl-puromycin) incorporation assay quantification in WT, $TSC2^{cntrl}$ & $TSC2^{mut}$ NSCs treated with IFN-β at 2 and 16 h. Two-way ANOVA with Dunnett's multiple comparison test was computed (*P*-values specified). $n = 5$ biological replicates for WT NSCs and 2–3 clonal replicates for $TSC2^{cntrl}$ & $TSC2^{mut}$ NSCs, respectively.

D Relate label-free quantification (LFQ) of proteomics from WT NSCs untreated or treated with IFN-β for 2 or 16 h. $n = 5$ biological replicates.

E OPP (O-propargyl-puromycin) incorporation assay quantification in WT NSCs treated with IFN-β at 16 h in the presence or absence of the integrated stress response inhibitor (ISRIB). One-way ANOVA with Tukey's multiple comparison test was computed (*P*-values specified). $n = 3$ biological replicates.

F Polysome profiling (RT-qPCR) of genes displaying upregulation of footprints in the ORF (Trex1 and Ifit2) compared to genes with an upregulation in uORFs (Slfn9 and Rbms2) upon 14 h IFN-β treatment. Hyphens represent mean of biological replicates. Arrows indicate the 40S, 60S and 80S subunits of the ribosome. Two-way ANOVA with Šídák's multiple comparison test was computed. Trex1 fraction 1 **($P = 0.0019$), fractions 2, 6, 7, 9, 10 ***($P < 0.0001$), fraction 5 **($P = 0.0016$). Ifit2 fractions 3, 8, 11 ***($P < 0.0001$), fraction 7 **($P = 0.0044$), fraction 9 **($P = 0.0031$), fraction 10 **($P = 0.0063$). Slfn9 fraction 6 *($P = 0.0357$). Outliers of fraction 1 from Ifit2, Slfn9 and Rbms2 were excluded from the statistical analysis. $n = 3$ biological replicates.

G Representative WB image and quantification (log2FC) of p-$TSC2^{Ser1452}$ from IFN-β-treated NSCs normalised to control ($t = 0$ h). Bars represent the mean. One sample *t*-test was computed. One hour *($P = 0.017$), 2 h **($P = 0.00565$), 16 h *($P = 0.0268$). $n = 3$ biological replicates.

Data information: $P \equiv P$-value.

© 2023 The Authors

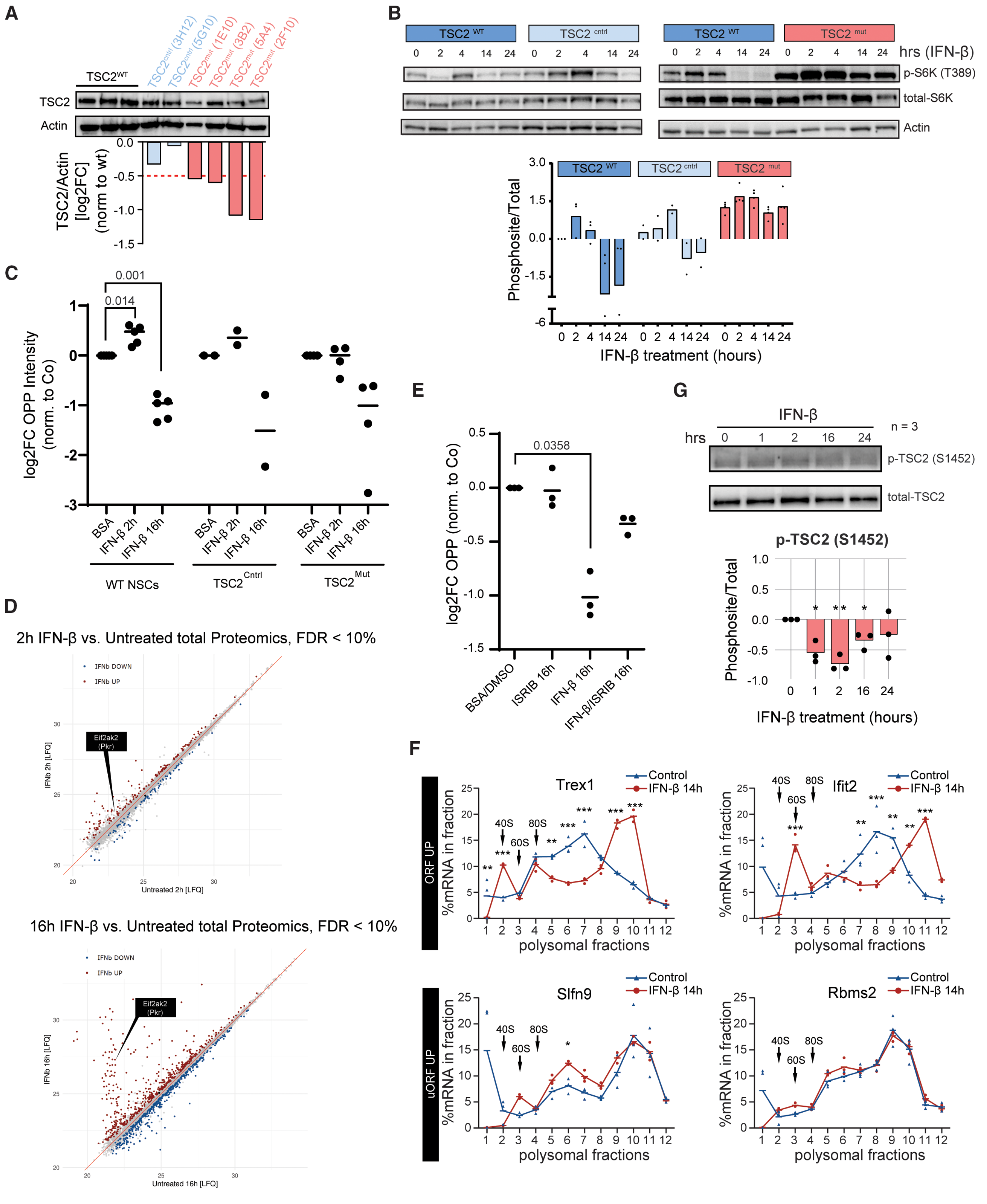

Figure EV4.

© 2023 The Authors

Figure EV5. Effect of interferon on Sox2 translation and modelling of interferon receptors removal along lifespan of animals.

A Representative WB image and quantification (log2FC) of Sox2 from IFN-β-treated NSCs normalised to control (*t* = 0 h). Bars represent the mean value. One sample *t*-test was computed. Sixteen hours *(*P* = 0.0219), 24 h **(*P* = 0.03), 48 h (*P* = 0.056). *n* = 4 biological replicates.

B 5'UTR constructs priming *renilla* luciferase controlled by the upstream 5'UTR fragment from Sox2, Rps21 and Actb. TOP = 5'Terminal Oligopyrimidine motif; PRM = 5' Pyrimidine Rich Motif.

C Luciferase activity assay in NSCs treated with Torin1. Data are normalised to vehicle and are represented as mean ± SD. *n* = 3 biological replicates. Two-way ANOVA test with Šídák's multiple comparison test (*P*-values specified).

D Luciferase activity assay in NSCs treated with Rapamycin (Rapa) and IFN-β. Data are normalised to vehicle and are represented as mean ± SD. *n* = 7 biological replicates. Two-way ANOVA test with Šídák's multiple comparison test (*P*-value specified).

E Luciferase activity assay in NSCs treated with Rapamycin. Data are normalised to vehicle and are represented as mean ± SD. *n* = 7 biological replicates. Two-way ANOVA test with Šídák's multiple comparison test (*P*-values specified).

F Luciferase activity assay in NSCs treated with the integrated stress response inhibitor (ISRIB) and IFN-β. Data are normalised to vehicle and are represented as mean ± SD. *n* = 5 biological replicates. Two-way ANOVA test with Šídák's multiple comparison test (*P*-values specified).

G Induced interferon knockout simulations (coloured, each colour represents a different intervention timepoint) of relative loss of stem cells (upper panel) across age compared to wildtype simulations (black; 100%). The relative rate (lower panel) at which progenitors are produced $(2.25emp_{s}.25em(1-b).25emaNSC)$ from NSCs for induced interferon knockout simulations with interferon dependent self-renewal compared to WT simulations. Dashed red line denotes age 350 days.

Data information: *P* ≡ *P*-value.

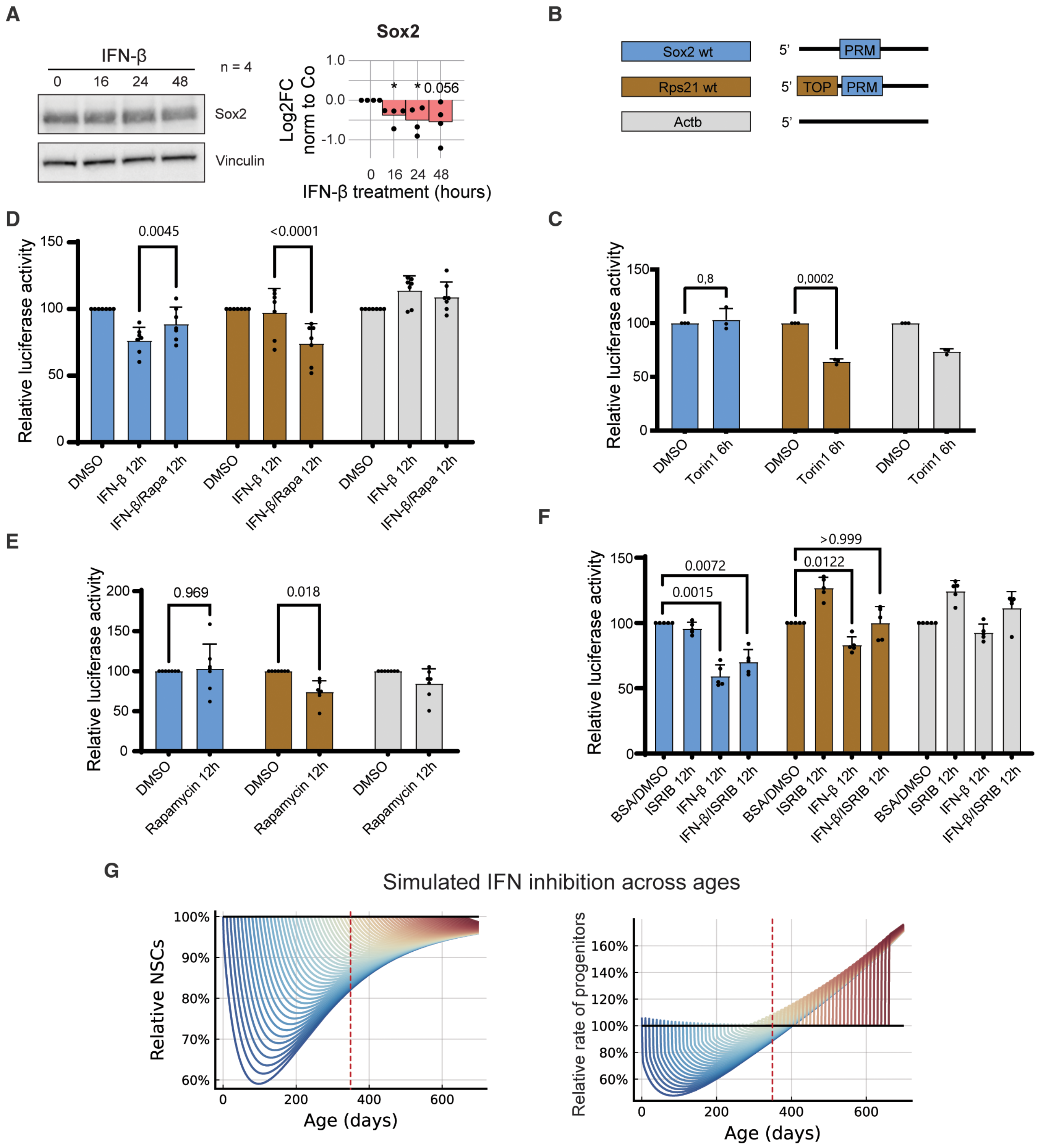

Figure EV5.

 © 2023 The Authors
