# Supplementary material for: Interferon regulates neural stem cell function at all ages by orchestrating mTOR and cell cycle
Source: EMBO Mol Med. 2023 Jan 13;15(4):e16434. doi: 10.15252/emmm.202216434 (PMC10086582; doi:10.15252/emmm.202216434)
Supplement: Supplementary file 9 [file EMMM-15-e16434-s004.pdf]

EMBO Molecular Medicine

Interferon regulates neural stem cell function at all ages by orchestrating mTOR and cell cycle

Damian Carvajal Ibañez, Maxim Skabkin, Jooa Hooli, Santiago Cerrizuela, Manuel Goepferich, Adrien Jolly, Katrin Volk, Marc Zumwinkel, Matilde Bertolini, Gianluca Figlia, Thomas Hoefer, Günter Kramer, Simon Anders, Aurelio Teleman, Anna Marciniak-Czochra, and Ana Martin-Villalba
DOI: 10.15252/emmm.202216434

Corresponding authors: Ana Martin-Villalba (a.martin-villalba@dkfz-heidelberg.de) , Anna Marciniak-Czochra (anna.marciniak@iwr.uni-heidelberg.de)

Review Timeline:

Submission Date:	12th Jun 22
Editorial Decision:	6th Jul 22
Revision Received:	21st Nov 22
Editorial Decision:	14th Dec 22
Revision Received:	15th Dec 22
Accepted:	16th Dec 22

Editor: Jingyi Hou

Transaction Report:

(Note: With the exception of the correction of typographical or spelling errors that could be a source of ambiguity, letters and reports are not edited. Depending on transfer agreements, referee reports obtained elsewhere may or may not be included in this compilation. Referee reports are anonymous unless the Referee chooses to sign their reports.)

6th Jul 2022

Dear Ana,

Thank you again for submitting your work to EMBO Molecular Medicine. We have now heard back from two of the three referees who agreed to evaluate your manuscript. As you will see from the reports below, the referees acknowledge the potential interest of the study. However, they raise a series of concerns, which we would ask you to address in a revision of the manuscript.

Without reiterating all the points raised in the reviews below, some of the more substantial issues are the following:

1. Considering Referee #3's comment on the lack of cohesion among various findings, please revise the manuscript to make the key conclusions more accessible to the general audience of EMBO Mol Med.

2. Both Referees (especially Referee #3) mentioned that some of the conclusions are not fully supported by the data, which must be carefully addressed. The referees recommended experiments in this regard, and the related statements need to be carefully worded to avoid overstatements.

We would welcome the submission of a revised version within three months for further consideration. Please note that EMBO Molecular Medicine strongly supports a single round of revision. As acceptance or rejection of the manuscript will depend on another round of review, your responses should be as complete as possible.

EMBO Molecular Medicine has a "scooping protection" policy, whereby similar findings that are published by others during review or revision are not a criterion for rejection. Should you decide to submit a revised version, I do ask that you get in touch after three months if you have not completed it to update us on the status.

We are aware that many laboratories cannot function at full efficiency during the current COVID-19/SARS-CoV-2 pandemic and have therefore extended our "scooping protection policy" to cover the period required for a full revision to address the experimental issues. Please let me know should you need additional time, and also if you see a paper with related content published elsewhere.

Please read below for important editorial formatting and consult our author's guidelines for proper formatting of your revised article for EMBO Molecular Medicine.

I look forward to receiving your revised manuscript.

Kind regards,
Jingyi

Jingyi Hou
Editor
EMBO Molecular Medicine

***** Reviewer's comments *****

Referee #1 (Remarks for Author):

In this work, Martin-Villalba and co-authors studied how INF signaling regulates NSC function in young and old brains. They first used scRNA-seq to compare the transcriptomes of NSC and their progeny in WT and INF receptor KO mice. They found that INF regulates NSC in both young and old brains, and INF receptor KO induces intrinsic INF signaling. Next, they studied the translational regulation of INF in NSCs and identified a biphasic mTORC1 activity that downregulates SOX2 level and induces cell cycle exit. In general, based on their previous breakthrough, the authors systematically revealed the importance of INF signaling in NSC and suggested a new therapeutic strategy to repair the aging brain based on a timely INF intervention. Overall, this work is well designed and the manuscript fits well with the scope of the journal.
I only found several points that the authors may need to improve or consider:
1, Please show p values and the IB confirmation (esp. p-AKT-S473) for Appendix Table 1.

2, From the text and the figure, I couldn't find a clear description for the TSC2 mut cells. Are there 4 clones and the first three are heterozygous and the fourth is homozygous? And which clone was used in EV3D? Obviously, IB in EV4A was not properly done, making it hard to judge whether the Tsc2 KO works. Please provide IB with all samples on the same blot.

3, For the mTOR inhibition in Fig3H, because Fig2I shows activation of S6K-S6 but not 4EBP pathway, and whether mTORC2 is involved is not clear, Rapamycin is expected to be used rather than Torin1. Or at least the authors should also try Rapamycin to see whether the same effect as Torin1.

4, As for the late phase of translational change, the evidence the authors provided may not be strong enough to support causal effects from mTORC1 feedback inhibition or eIF2a activation. Maybe this was shown in their previous paper, but I wonder whether the authors could intervene the two regulators separately and in combination at the late phase and detect both global and specific gene translations such as Rps21 and Sox2.

Referee #3 (Comments on Novelty/Model System for Author):

Suggestions are included in the critique

Referee #3 (Remarks for Author):

Carvajal Ibañez et al describe an age-dependent biphasic role for interferon-β in cell cycle progression of stem cells and mTORC1 signaling regulation. Specifically, the authors show that interferon-β treatment arrests stem cells in G0 of the cell cycle and attenuates their mTORC1 signaling following an acute increase. The attenuated mTORC1 activity correlates with a global

reduction in protein synthesis, reduced translational efficiency of TOP mRNAs, and reduced Sox2 translation, which allows for differentiation. NSCs from mice lacking IFNAR and IFNGR lack the age-dependent differential IFN-β response, suggesting an important role of IFN signaling in NSCs during aging.

General comment:

The authors combine several mouse models and omics approaches to produce interesting findings. While the importance of an intrinsic interferon program has been previously identified in stem cells (Wu et al 2008), biphasic control of mTORC1 and global translation by interferon in NSCs is novel. Some claims are not fully supported by data and there is a lack in cohesion among the various findings, rendering the main conclusions of the study confusing. For example, the authors claim, "Interferon orchestrates cell cycle and mTOR activity to post-transcriptionally repress Sox2 and drive the exit from stem cell activation." However, there are no experiments in the paper that address "the exit from stem cell state". Also, the link between the biphasic mTORC1 activity in stem cell aging and aging in animals is tenuous.

Specific comments:

1-The title is misleading by referring to "stem cell" while it only describes studies with NSCs. It refers to "at all ages", while only two age groups were studied. Some terms that are used in the manuscript are not clearly defined and confusing:

- "interferon signaling in stem cells also reduces their ability to activate. "To activate what?
- "the molecular link between interferons and activation in neural stem cells and how this relates to productivity is not well understood." What is meant by "productivity"?

2-The introduction is not adequate and needs elaboration. The main problem and the hypothesis are not clear. It would be helpful to include a summary of the findings.

3-The schematics in Fig. 1d are not intuitive and poorly descripted in the figure legends.

4-The switch between IFNRA or IFNAR, and IFNRG or IFNGR in the text is confusing.

5- Changes in global translation observed in Figs. 2A and B and Figs. EV4B, and C need to be strengthened either by quantification of the area under the polysomes or by use of a more quantitative assay such as 35S-Met or puromycin incorporation.

6- The authors need to provide some assessment of the quality of their ribosome profiling data such as replicate correlation, etc.

7- It is unclear why the authors have used LT-test for calculating FDR for the ribo-seq experiments, there are many tools such as deltaTE, Anota2seq with established built-in feature to calculate FDR.

8- Figs. 2E and F; the authors should provide the criteria they used to define TOP mRNAs and the list of TOP mRNAs they assessed in these figures. Do any of the TOP mRNA in ribosome profiling data reach statistical significance? It appears that for many of these genes there is not a significant change in Riboseq level. It has been reported that TOP mRNAs are regulated by LARP1, is there evidence of LARP1 activity in this setting?

9- Fig. 2i; It is not clear why the authors checked 4E-BP phosphorylation at S65 as opposed to the other phosphorylation sites. Please specify if this is this 4EBP1 or 4EBP2.

10- Figs. 2i and Fig. 3a; the composite blots are clearly not all derived from the same gel/membrane. The authors should either show composites belonging to the same gel/membrane, or clearly label which composites correspond to the same gel/membrane.

11- Figs. 3D and EV4F: Is there an increased in local 5'UTR footprint density in a subset of ISGs including Ifit3 and Irf9?

12- Fig. S3a: Blots for WT and Mut tissue must be on the same membrane to ensure same transfer of protein and exposure time during acquisition.

13- Figs. 3e and f: Can the authors provide direct evidence that TSC1/2 is being regulated by Cdk4/6 as proposed? Perhaps a western for TSC2 Ser1452 and Ser1217.

14- Authors need to show Sox2 protein levels to confirm that the transition of an activated stem cell into a differentiated neuroblast is mediated by Sox2 and controlled by IFN type I.

15- In the discussion, the wording of the following statements needs to be changed: "Our study shows that in the natural environment of the brain, the ISGs expressed upon exposure to Type I interferon in NSCs ex-vivo...". This is not correct since ex-vivo is not the same as the natural environment of the brain nor is the experimental manipulation of IFN-β levels.

Point-by-point responses to Review of EMM-2022-16434

Referee's reports and point-by-point responses

We thank both Referees for the constructive feedback and their willingness and time to consider our manuscript. Referees have highlighted the interest and well-fitting scope of our results to EMBO Mol Med and have shown appreciation of our combination of omics in well-designed experiments – commenting on being "well designed and the manuscript fits well with the scope of the journal" (Referee #1). While the importance of an intrinsic interferon program has been previously identified in stem cells (Wu *et al*, 2018), biphasic control of mTORC1 and global translation by interferon in NSCs is novel (Referee #3).

At the same time, Referees have raised two main concerns:

1. Both Referees find some claims not being fully supported by the data.
2. Referee #3 finds that there is a lack of cohesion leading to confusing main conclusions.

Following their suggestions, we run a substantial number of new experiments and analyses to address their comments. These are found below as well as implemented in the revised manuscript. Referee's comments are coloured in orange and changes in the main text of the manuscript are highlighted in blue.

Referee #1 (Remarks for Author):

In this work, Martin-Villalba and co-authors studied how INF signaling regulates NSC function in young and old brains. They first used scRNA-seq to compare the transcriptomes of NSC and their progeny in WT and INF receptor KO mice. They found that INF regulates NSC in both young and old brains, and INF receptor KO induces intrinsic INF signaling. Next, they studied the translational regulation of INF in NSCs and identified a biphasic mTORC1 activity that downregulates SOX2 level and induces cell cycle exit. In general, based on their previous breakthrough, the authors systematically revealed the importance of INF signaling in NSC and suggested a new therapeutic strategy to repair the aging brain based on a timely INF intervention. Overall, this work is well designed and the manuscript fits well with the scope of the journal.

We thank Referee #1 for their time and appreciation of the scientific quality of this work and its relevance for publication in EMBO Mol Med.

I only found several points that the authors may need to improve or consider:

1, Please show p values and the IB confirmation (esp. p-AKT-S473) for Appendix Table 1.

Thanks for pointing this out. We now provide p-values for Appendix Table 1 (now Table EV1) and assessed the differential phosphorylation levels of p-Akt^{S473} in NSCs upon IFN-β treatment by western-blot.

Table EV1: List of relevant phosphosites.

Protein	Phosphosite	log2FC (2h)	Student's T-test p-value 2h	log2FC (16h)	Student's T-test p-value 16h
Akt1	S473	0.1	0.8889484970325127	-1	0.01947421677517165
Irs1	Y608	1.2	0.2320133634496766	0.3	0.7793867172013139
Irs1	S1134	0.9	0.042653040984653716	-2.3	0.15171237251216912
Irs1	S1137	0.2	0.8769927437047635	-1.7	0.39230243158524875
Eif2ak2	S32	0.1	0.8175234144270087	4.2	1.1782026629913688e-4
Eif2ak2	S110	0.4	0.5615209963409157	3.3	9.414125855447387e-4
Eif2ak2	S163	-0.2	0.6399705659264385	2.4	2.9907492328004408e-5
Eif2ak2	S223	0.5	0.29607517864818905	2.1	9.135240808271367e-4
Eif2ak2	S240	-1	0.14584414540390048	3.1	0.023779898687111937
Ccnd1	T286	1.9	0.09877794801291162	0.3	0.679072275691531
Larp1	S498	1	0.13789801033756613	3.2	4.3279277291754484e-4
Larp1	T492	-0.1	1	2.6	0.021983660069913993

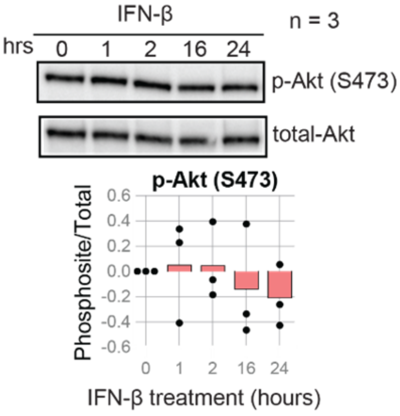

We find that the mTORC2-dependent phosphosite p-Akt^{S473} is not affected by IFN-β treatment in NSCs. This shows that IFN-β treatment in our system targets mTORC1 selectively. Additionally, we validated this selective response by testing the effect of rapamycin vs. Torin1 inhibition as suggested by this Referee as well (see detailed point-by-point comment below).

These results can be found in the adapted Table EV1, Fig EV3F and the main text as:

Page 7: Notably, the mTORC2-dependent p-Akt^{Ser473} site remained unaffected by IFN-β treatment (Fig EV3F). This indicates that the early crosstalk between IFN-β and the PI3K pathway upregulates mTORC1 selectively, with no effect on mTORC2 at any time of interferon exposure in NSCs.

2, From the text and the figure, I couldn't find a clear description for the TSC2 mut cells. Are there 4 clones and the first three are heterozygous and the fourth is homozygous? And which clone was used in EV3D?

We now added identifiers to the individual clones to ease the matching between different experiments and clones in Fig EV4A and Appendix III.

Regarding the description of mutant cells; $TSC2^{mut}$ NSC clones were defined as those NSCs where the protein expression of TSC2 was reduced to at least half compared to the wildtype NSCs. As we could not detect a complete loss of TSC2 protein, we define these clones as heterozygous mutant. A more detailed description is now implemented in the main text as:

Page 8: To validate this, we generated four heterozygous TSC2 mutant NSC clones by CRISPR/Cas9 which halved their levels of TSC2 (labelled $TSC2^{mut}$). In addition, two NSC clones where CRISPR/Cas9 did not reduce TSC2 protein levels were used as control (labelled $TSC2^{ctrl}$) to exclude off-target or manipulation-derived artefacts (Fig EV4A).

Obviously, IB in EV4A was not properly done, making it hard to judge whether the Tsc2 KO works. Please provide IB with all samples on the same blot.

We now repeated and updated the blot showing all controls and clones in the same blot in Fig EV4A:

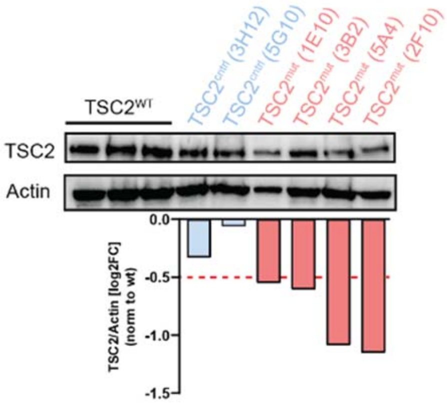

3, For the mTOR inhibition in Fig3H, because Fig2I shows activation of S6K-S6 but not 4EBP pathway, and whether mTORC2 is involved is not clear, Rapamycin is expected to be used rather than Torin1. Or at least the authors should also try Rapamycin to see whether the same effect as Torin1.

We focussed on Torin1 because, differently to rapamycin, Torin1 fully inhibits mTORC1 activity (Thoreen *et al*, 2012). Now, following the recommendation of Referee #1 we assessed the effect of rapamycin. We observe that both rapamycin and Torin1 exert similar effects, confirming that the inhibition of Sox2 translation by IFN-β is mTORC1-dependent.

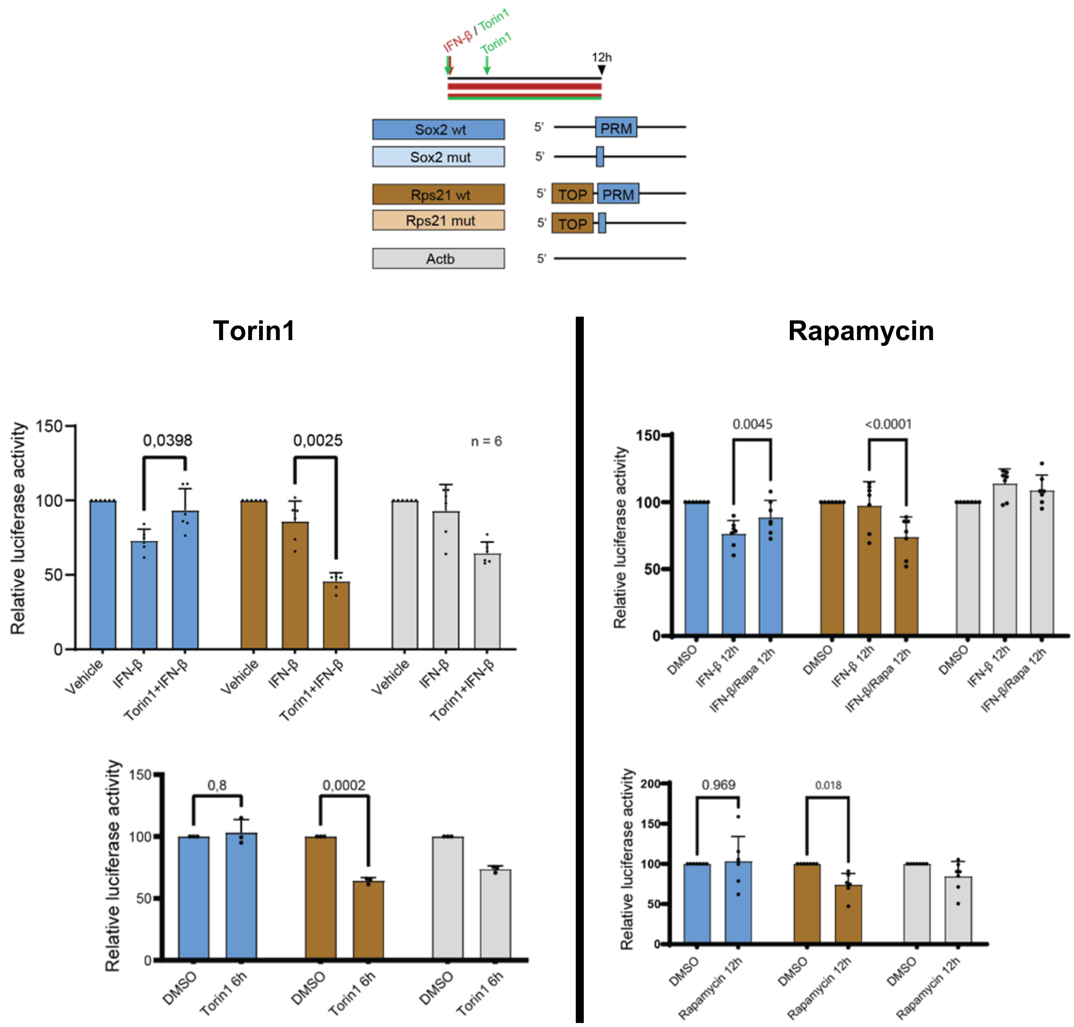

These results are provided in Fig EV5 and are implemented in the main manuscript as:

Page 10: We decided to use Torin1 as, differently to rapamycin, it fully inhibits mTORC1 activity (Thoreen *et al*, 2012). However, we still observe the same results using rapamycin (Fig EV5D,E) confirming the selective relevance of mTORC1 in the IFN-β response in NSCs.

4, As for the late phase of translational change, the evidence the authors provided may not be strong enough to support causal effects from mTORC1 feedback inhibition or eIF2a activation. Maybe this was shown in their previous paper, but I wonder whether the authors could intervene the two regulators separately and in combination at the late phase and detect both global and specific gene translations such as Rps21 and Sox2.

The presence of inhibitory feedback loops upon mTORC1 activation acting upstream of IRS1/2-Akt are already described in the literature (Hsu *et al*, 2011). We now clarified this in the main manuscript including the above-mentioned reference.

For exclusive eIF2a inhibition, we now blocked the effect of increased p-eIF2α^{Ser51} upon IFN-β treatment by co-incubation with the Intrinsic Stress Response Inhibitor (ISRIB) (Sidrauski *et al*, 2015). We observe that blocking the ISR in combination with IFN-β reduced the late downregulation of global protein translation, assessed by the incorporation of OPP in IFN-β-treated NSCs. This confirms the contribution of eIF2α to the late repression of protein synthesis in combination with the late inhibition of mTORC1 exerted by IFN-β.

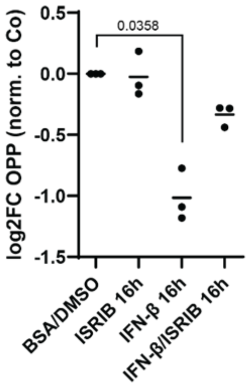

As blockage of ISR reversed the global translation shutdown, it also rescued the inhibition of *Rps21* translation upon IFN-β treatment. Strikingly, blocking ISR does not affect translation control of *Sox2*, confirming the selective relevance of mTORC1 to the IFN-β-mediated repression of *Sox2* translation.

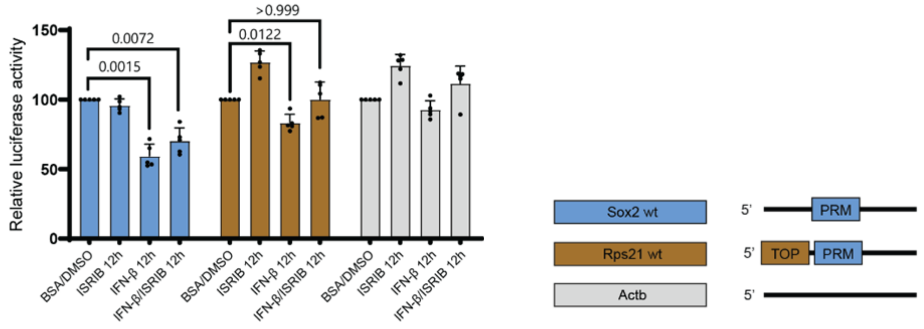

These results are provided in Fig EV2D,E, EV4E and EV5F and are implemented in the main manuscript as:

Page 8: Indeed, selective inactivation of the p-eIF2α^{Ser51} response by the Intrinsic Stress Response Inhibitor (ISRIB) (Sidrauski *et al*, 2015) reduced the late downregulation of global protein synthesis in IFN-β-treated NSCs (Fig EV4E).

Page 10: In addition, given the effect of increased p-eIF2α^{Ser51} in protein synthesis in the IFN-β response in NSCs (Fig 3C), we wondered if p-eIF2α would also influence Sox2 translation. To test this, we blocked the effect of p-eIF2α^{Ser51} using ISRIB (Sidrauski *et al*, 2015). Blocking the ISR in the presence of IFN-β restored global translation levels (Fig EV4E) and rescued the repression of translation of *Rps21*, but failed to significantly affect Sox2 translation (Fig EV5F).

Referee #3 (Comments on Novelty/Model System for Author):

Suggestions are included in the critique

Referee #3 (Remarks for Author):

Carvajal Ibañez et al describe an age-dependent biphasic role for interferon-β in cell cycle progression of stem cells and mTORC1 signaling regulation. Specifically, the authors show that interferon-β treatment arrests stem cells in G0 of the cell cycle and attenuates their mTORC1 signaling following an acute increase. The attenuated mTORC1 activity correlates with a global reduction in protein synthesis, reduced translational efficiency of TOP mRNAs, and reduced Sox2 translation, which allows for differentiation. NSCs from mice lacking IFNAR and IFNGR lack the age-dependent differential IFN-β response, suggesting an important role of IFN signaling in NSCs during aging.

General comment:

The authors combine several mouse models and omics approaches to produce interesting findings. While the importance of an intrinsic interferon program has been previously identified in stem cells (Wu et al 2008), biphasic control of mTORC1 and global translation by interferon in NSCs is novel.

We thank Referee #3 for praising our combination of animal models and omics as well as the novel finding of control of mTORC1 by IFN. We also thank this referee for suggestions and constructive criticism of our manuscript.

Some claims are not fully supported by data and there is a lack in cohesion among the various findings, rendering the main conclusions of the study confusing.

To improve clarity on the comments from Referee #3, we would like to raise our concern on the "lack of cohesion" found by Referee #3. We suspect that many suggestions and criticism from Referee #3 may not apply to the submitted version of the manuscript but to the review of a previous version. Between these two versions, we adapted the whole manuscript to the demands of cohesion and clarity of our hypotheses and conclusions that Referee #3 reiterated here. We are therefore unsure whether Referee #3 still found these concerns in the new version, since sometimes parts that do not exist in the current version are referred to. In addition, most new concerns from Referee #3 refer only to the title or the abstract. Examples of this comments are disclaimed over the point-by-point and the rest of raised points are addressed below.

For example, the authors claim, "Interferon orchestrates cell cycle and mTOR activity to post-transcriptionally repress Sox2 and drive the exit from stem cell **activation**." However, there are no experiments in the paper that address "the exit from stem cell state".

We thank Referee #3 for raising this concern. Indeed, we claim that interferon drives the exit of stem cell **activation**, but not of stem cell state/function. We would like to discuss the importance of differentiating these two terms and highlight that our claim refers to the exit of stem cell **activation**:

- Active cell cycle is one of the main markers to define activation in stem cells (Cho *et al*, 2019). We show that interferon inhibits cell cycle in actively-cycling NSCs ex-vivo and keeps NSCs in the G0 (quiescent) cell cycle state (Fig 1C and EV2). Ribo-Seq of interferon-treated NSCs also shows that interferon downregulates the translation efficiency of cell cycle checkpoint genes (Fig EV3B). In addition, we show that interferon treatment inhibits Cdk4/6, the main regulator of the G1/S cell cycle checkpoint, in NSCs (Fig 3E).
- High translation is a main feature of activation of stem cells in general and of NSCs in particular. Translation levels are strongly reduced in quiescent NSCs and mildly reduced upon differentiation (Llorens-Bobadilla *et al*, 2015; Baser *et al*, 2019). We show that interferons induce a bi-phasic control of mRNA translation to strongly reduce translation levels in active NSCs (Fig 2A-B,E-F; EV3D). In addition, we now performed OPP incorporation assays confirming that IFN-β shuts down global protein synthesis in NSCs (Fig EV2D,E), which indicates the exit of an activation state (fully-detailed below in a response to Referee #3).
- Sox2 is a well-known marker of stem cells and it has been described to be reduced in quiescence and upon differentiation of NSCs (Baser *et al*, 2019; Marqués-Torrejón *et al*, 2013). We show that interferon inhibits expression of Sox2 post-transcriptionally in NSCs (Fig 3G-J and EV4G). In addition, we now showed that IFN-β drives a mild reduction of Sox2 protein (Fig EV5A) (fully-detailed below in a response to Referee #3).

Given that we show that interferon modulates these hallmarks of stem cell activation, we find our claim that "Interferon orchestrates cell cycle and mTOR activity to post-transcriptionally repress Sox2 and drive the exit from stem cell activation" well-grounded.

Also, the link between the biphasic mTORC1 activity in stem cell aging and aging in animals is tenuous.

We would like to highlight the fact that our results show that interferon type I induces a biphasic response of mTORC1 in **active stem cells** (ex-vivo). mTOR activity is cell cycle dependent in NSCs and quiescent NSCs have the lowest mTOR activity in-vivo (Romero-Pozuelo *et al*, 2020). Active NSCs are mostly present in young individuals, as opposed to ageing where quiescence NSCs become predominant (Kalamakis *et al*, 2019). Our results point out that interferon activity is already present at young ages and that the exit of stem cell activation by interferon contributes to the increased fraction of quiescent cells in the aging brain -as shown with our mathematical modelling-. In brief, we have shown with scRNAseq from animal models, quantifications of active and quiescent NSCs in-vivo and mathematical modelling that interferon is modulating stem cell function along the lifespan of individuals (Fig 1A,B,F-G; Fig4; EV1 and EV5). In particular, we show that interferons regulate the number of stem cells by increasing quiescence, i.e. reducing the rate of activation, and increasing the probability of self-renewal of NSCs. The former can be explained by the interferon-mediated exit of stem cell activation as discussed above, linking the biphasic mTORC1 with the response of NSCs *in-vivo* in the absence of interferon.

The link between exit of activation state by the biphasic control of mTORC1 and aging is now better described in the discussion as:

Page 16: This exit from activation after a life-long exposure to interferons results in a more quiescent old brain, when interferon signalling is found at highest.

Specific comments:

1-The title is misleading by referring to "stem cell" while it only describes studies with NSCs. It refers to "at all ages", while only two age groups were studied.

We thank the referee for these remarks. We now added “neural” to the title.

While it is true that in our single-cell sequencing we only studied two ages (we removed the “at all ages” from the title from Fig1), our mathematical modelling which makes broader statements about neurogenesis was parameterised on data obtained from mice aged 2 months, 6–7 months and 22 months. These ages were chosen so they cover the typical lifespan of an animal. Our mathematical model allows us to infer dynamics for these specific times in between these times as well, thus giving statements about **neurogenesis at all ages** (see Appendix supplementary methods).

Some terms that are used in the manuscript are not clearly defined and confusing:

- "interferon signaling in stem cells also reduces their ability to activate. "To activate what?

Activation of stem cells or the ability of a stem cell to activate is defined as the exit of a quiescent or resting stem cell state to divide either in a committed/differentiated cell or another NSC (self-renewal). Relevant literature has referred to the “activation” of stem cells across different tissues in the past (Heidt *et al*, 2014; Almada *et al*, 2021; Urbán & Cheung, 2021). We now re-phrased this claim in the abstract to make it clear that it refers to activation of stem cells as:

Page 2: In the ageing brain, interferon signalling also reduces the ability of stem cells to activate.

- "the molecular link between interferons and activation in neural stem cells and how this relates to productivity is not well understood." What is meant by "productivity"?

We kindly apologise for the confusion generated by this term. We now replaced the term “productivity” for “progenitor production” in the manuscript. Alternatively, we would also propose the term progenitor output if Referee #3 would still have concerns about the “production” term.

Page 2: Additionally, the molecular link between interferons and activation in neural stem cells and how this relates to progenitor production is not well understood.

Page 12: Young IFNAGR KO mice have a lower activation rate of NSCs than WT, suggesting a decreased progenitor production. Older IFNAGR KO mice however have a higher activation rate than WT, suggesting increased progenitor production (Fig 4C).

Page 13: Overall, this shows that there may be benefits to progenitor production from blocking interferon response at increasing ages, while it may have strong adverse effects on short-term progenitor production and stem cell counts when intervening too early.

2-The introduction is not adequate and needs elaboration. The main problem and the hypothesis are not clear.

This suggestion from Referee #3 might not be based on the submitted version of the manuscript.

The introduction was already changed in the submitted version of the manuscript where we incorporated a more detailed description of the main problem and hypothesis of our research.

It would be helpful to include a summary of the findings.

We thank Referee #3 for this suggestion. A summary of the findings at the end of the introduction to facilitate comprehension to the readers is now added to the revised manuscript as:

Page 3: Here, single-cell RNA sequencing (scRNAseq) of IFNAGR^{KO} and wt NSCs reveals that already at young ages NSCs exhibit an IFN-response, as opposed to previous reports suggesting that this response is only found in aging NSCs (Kalamakis *et al*, 2019; Dulken *et al*, 2019; Baruch *et al*, 2014). Interestingly, already committed immature TAPs and neuroblasts are resilient to IFNs, revealing a hierarchy of interferon responsiveness along the stem cell lineage. To address the molecular underpinnings of NSCs IFN response, we performed Ribo-Seq in NSCs exposed to IFNs. Our data indicates that type-I IFN induces a transient up- and a late down-regulation of mTORC1 activity and a concomitant gradual inhibition of cell cycle. This biphasic control of mTORC1 is mediated by the crosstalk of the JAK-STAT and PI3K-Akt signalling pathways. In addition, late phosphorylation of eIf2α and Cdk4/6-mediated TSC2 inhibition contribute to the shutdown of mTORC1 and protein translation. Together, this IFN- response leads to the post-transcriptional inhibition of Sox2 expression. Mathematical modelling of NSC dynamics *in vivo* uncovers interferons as regulators of neural stem cell activation and self-renewal at all ages. Consequently, modelling predicts that inhibition of interferon is detrimental in the young and beneficial in the old brain.

3-The schematics in Fig. 1d are not intuitive and poorly descripted in the figure legends.

This suggestion from Referee #3 might not be based on the submitted version of the manuscript.

The scheme that Referee #3 criticised from the previous version is currently better described and placed in Fig EV2B.

4-The switch between IFNRA or IFNAR, and IFNRG or IFNGR in the text is confusing.

This suggestion from Referee #3 might not be based on the submitted version of the manuscript.

We could not find any “IFNRA” or “IFNRG” terms in the current version of the manuscript.

5- Changes in global translation observed in Figs. 2A and B and Figs. EV4B, and C need to be strengthened either by quantification of the area under the polysomes or by use of a more quantitative assay such as 35S-Met or puromycin incorporation.

We now applied OPP (O-propargyl-puromycin) incorporation experiments to quantitatively measure protein translation upon IFN-β treatment. In agreement with our observation on the polysome profiles, we find that IFN-β induces a transient mild increase followed by a profound inhibition of protein translation in WT and $TSC2^{cntrl}$ NSCs. In $TSC2^{mut}$ NSCs, early upregulation of protein synthesis is blunted, while the shutdown of protein production at late incubation remained. This denotes the PI3K-crosstalk and inhibitory feedback loops converging on TSC1/2 as the main drivers of the biphasic regulation of mTORC1, while unveiling additional components triggering the late protein synthesis shut-down exerted by IFN-β in NSCs. We now also assess the implication of p-eIF2α in this late downregulation in the revised manuscript, as suggested by Referee #1.

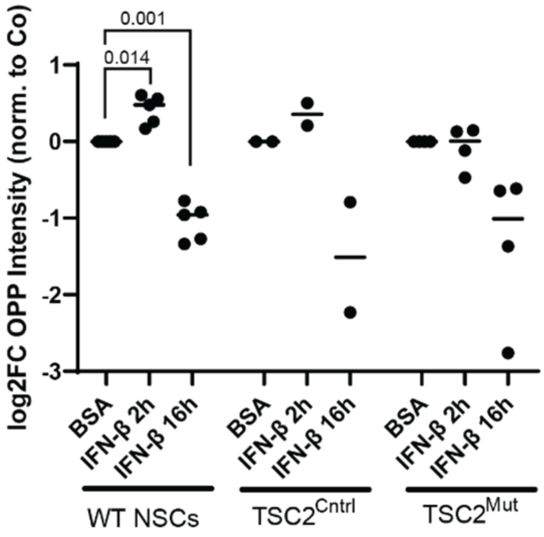

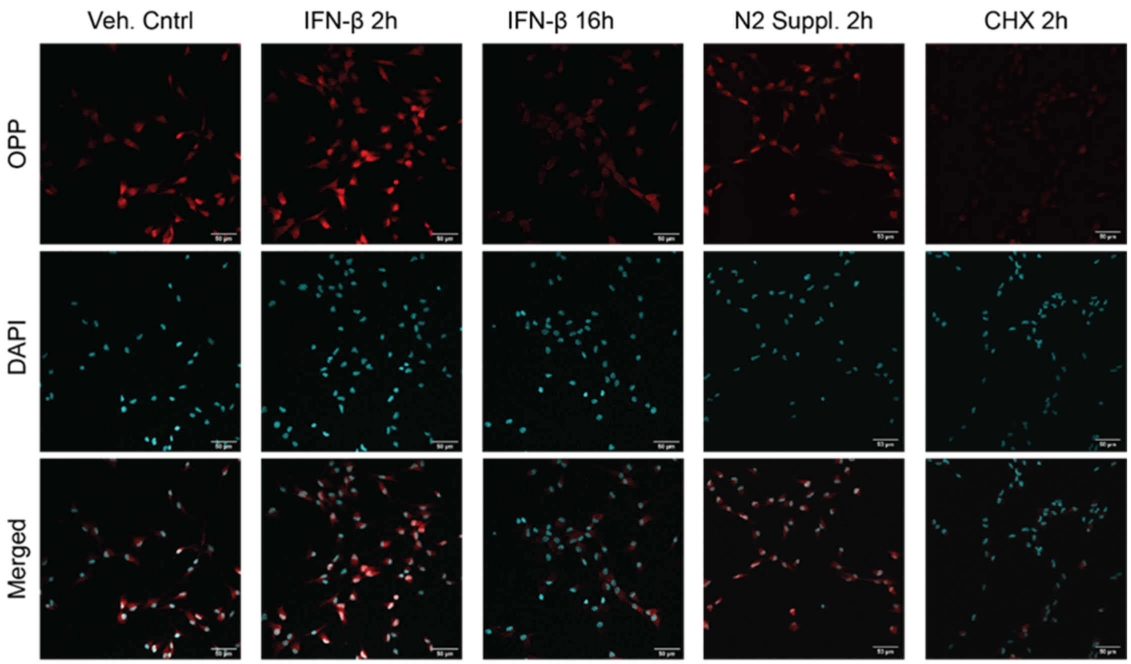

These results are provided in now Fig EV2D,E and EV4C and are implemented in the main manuscript as:

Page 5: Analysis of the polysome profiles of IFN-β-treated NSCs revealed a mild and transient increase followed by a strong decrease of the heavy polysomal fractions, as compared to untreated controls (Fig 2A,B). Accordingly, OPP (O-propargyl-puromycin) incorporation revealed a transient early increased followed by a profound decrease of global protein synthesis following exposure to IFN-β (Fig EV2D,E).

Page 8: Interestingly, the biphasic regulation of global protein synthesis was not detected in TSC2^{mut} NSCs. These mutant cells only exhibited a downregulation of global protein synthesis upon longer exposure to IFN (Fig EV4C). This uncovers the PI3K-crosstalk and inhibitory feedback loops converging on TSC1/2 as the main drivers of the early regulation, while unveiling additional contribution of eIF2α to the late shut-down.

6- The authors need to provide some assessment of the quality of their ribosome profiling data such as replicate correlation, etc.

We included now replicate correlation quality assessment in Appendix Figure S1:

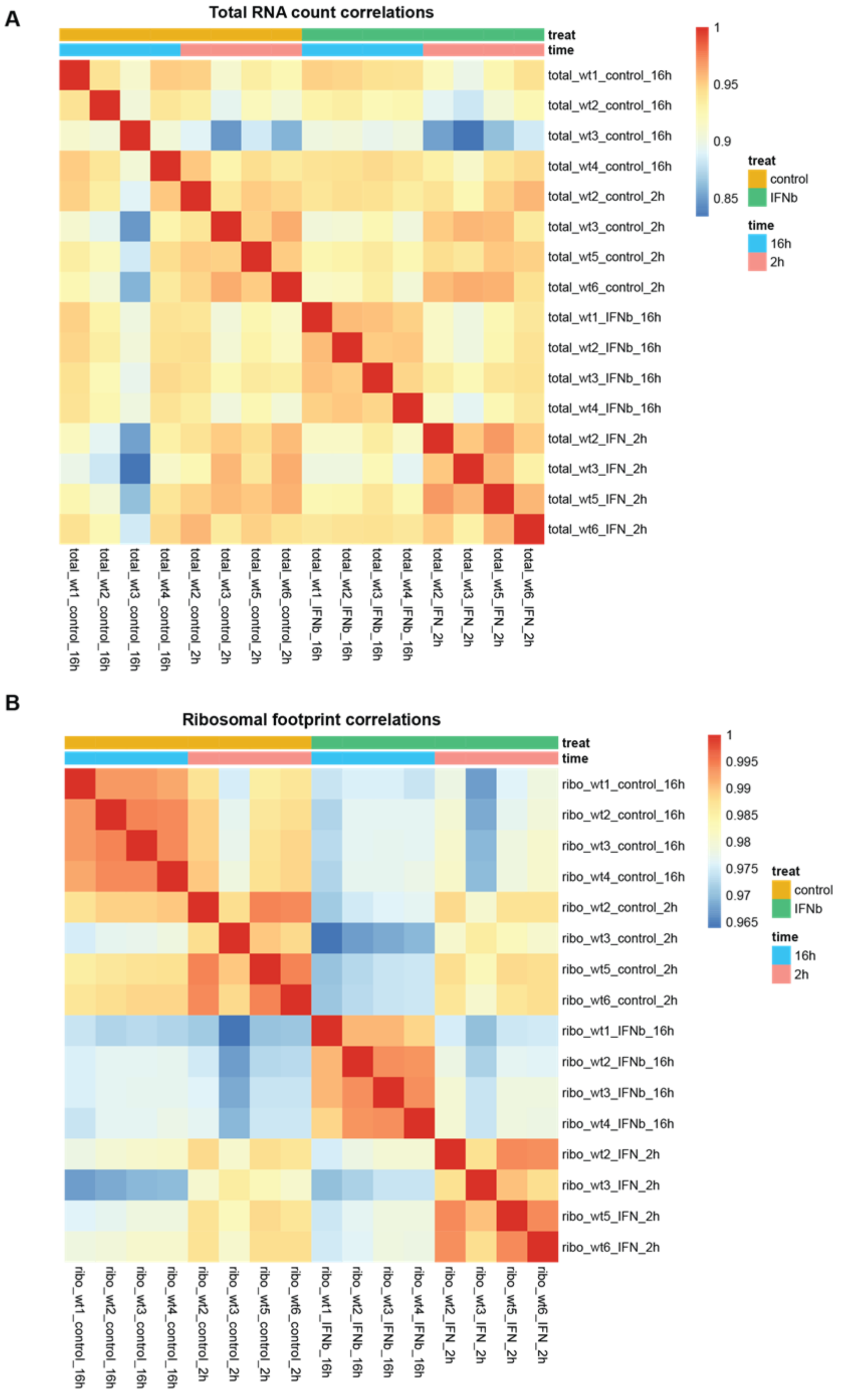

Appendix Figure S1. Ribo-Seq sample correlation quality control

A. Correlation matrix for ribosomal footprint libraries depicting pearson correlation coefficients for all samples. n = 4 biological replicates.

B. Correlation matrix for total RNA libraries depicting pearson correlation coefficients for all samples. n = 4 biological replicates.

7- It is unclear why the authors have used LT-test for calculating FDR for the ribo-seq experiments, there are many tools such as deltaTE, Anota2seq with established built-in feature to calculate FDR.

This suggestion from Referee #3 might not be based on the submitted version of the manuscript.

As written in our captions and methods we computed p-values with DESeq2 using a likelihood-ratio test and used multiple testing corrections supplied by DESeq2. This is considered best-practice for modelling count data from RNA sequencing experiments.

8- Figs. 2E and F; the authors should provide the criteria they used to define TOP mRNAs and the list of TOP mRNAs they assessed in these figures.

We defined TOP mRNAs “as those with a cytidine immediately after the 5′ cap, followed by an uninterrupted stretch of 4–14 pyrimidines” in agreement with previous publications (Thoreen *et al*, 2012). This is now implemented in the Methods section as:

Page 30: TOP-mRNAs (Table EV5) were defined as mRNAs with a cytidine immediately after the 5′ cap, followed by an uninterrupted stretch of 4–14 pyrimidines (Thoreen *et al*, 2012).

Do any of the TOP mRNA in ribosome profiling data reach statistical significance? It appears that for many of these genes there is not a significant change in Riboseq level.

We now had a closer look at the statistical significance of TOP-mRNAs in our Ribo-Seq. The computed Translation Efficiency (TE) and p-values are now provided as Table EV5. In this table the complete list of mRNAs defined as TOP-mRNAs is also provided to the readers. At IFN-β 2h, despite the footprint abundance observed in TOP-mRNAs, only 2 TOP-mRNAs reach statistical significance. We now changed our claims in the main text to a “tendency of an early up-regulation”, to be consistent with the statistical test results. This tendency is solid and reproducible as we also observe it in polysome profile-qPCRs of Rps17 and Rpl34, with statistical significance in the latter (Fig EV3D). In addition, the early upregulation of mTORC1 activity, which is the master regulator of TOP-mRNAs, is confirmed by WBs in Fig 2I.

At IFN-β 14h, together with the reduced footprint abundance observed in TOP-mRNAs, 17 TOP-mRNAs reach statistical significance. These results provide enough confidence to support our claim that TE of TOP-mRNAs is downregulated upon extended exposure to IFN-

β in NSCs. We reproducibly observe this downregulation also in polysome profile-qPCRs of Rps17 and Rpl34, where the dramatic repression is highly significant (Fig EV3D). In addition, repression of mTORC1 activity is confirmed by WBs in Fig 2I.

We implemented these changes in the revised manuscript as:

Page 6: We observed that IFN-β controlled TOP-mRNAs in a biphasic manner, exhibiting a tendency to early upregulation and a significant downregulation of translation index (Fig 2E,F and EV3D; Table EV5).

In addition, we reformatted Fig2E,F to display consistent axis as Fig2C,D:

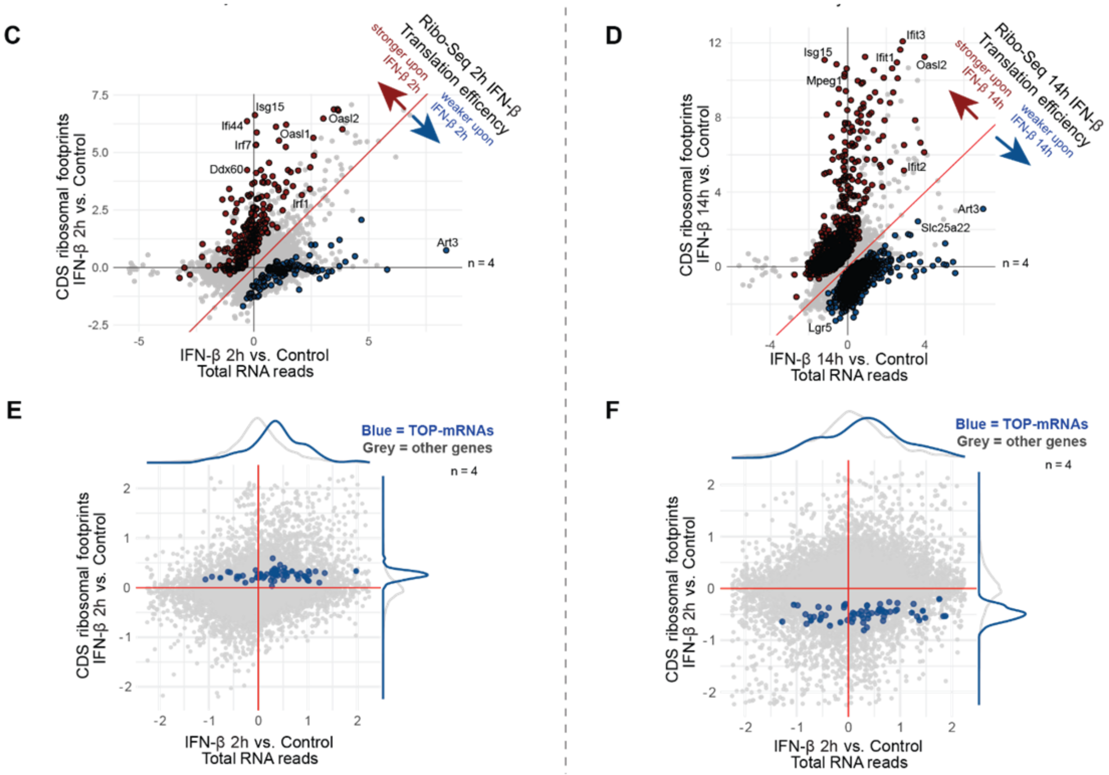

It has been reported that TOP mRNAs are regulated by LARP1, is there evidence of LARP1 activity in this setting?

We appreciate the interest of Referee #3 in the regulation of LARP1 in our system.

We assessed levels of LARP1 total protein by WB and could not detect any changes in NSCs exposed to IFN-β.

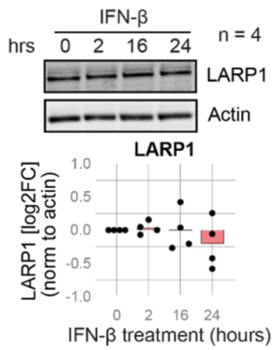

In addition, we checked phosphorylation of LARP1 in our phospho-proteome dataset. We found that extended IFN-β treatment led to a significant upregulation in phosphorylation of LARP1^{Ser498} (human Ser521) and LARP1^{Thr492} (human Thr515), both located in the RRM domain from the La module of LARP1 (Jia *et al*, 2021). Despite these findings, the biological relevance of these phosphosites are to date not described and therefore we cannot confidentially claim a change of LARP1 activity in our system.

Table EV1: List of relevant phosphosites.

Protein	Phosphosite	log2FC (2h)	Student's T-test p-value 2h	log2FC (16h)	Student's T-test p-value 16h
Larp1	S498	1	0.13789801033756613	3.2	4.3279277291754484e-4
Larp1	T492	-0.1	1	2.6	0.021983660069913993

These new findings are now implemented in Fig EV3E, Table EV1 and the revised version of the manuscript as:

Page 7: We additionally assessed whether LARP1, a key repressor of TOP-mRNA translation, would be regulated by IFN-β in NSCs. We find that p-LARP1^{Ser498} (human ^{Ser521}) and p-LARP1^{Thr492} (human ^{Thr515}) are differentially phosphorylated at 16h of IFN-β treatment, and no change in total LARP1 protein (Fig EV3E; Table EV1). However, as the biological relevance of these phosphosites is unclear (Jia *et al*, 2021), the role of LARP1 in regulation of TOP mRNAs translation in NSCs remains elusive.

9- Fig. 2i; It is not clear why the authors checked 4E-BP phosphorylation at S65 as opposed to the other phosphorylation sites. Please specify if this is this 4EBP1 or 4EBP2.

We examined 4E-BP1. This is now specified in the text and figures. We decided to check Ser65 as other phosphosites of 4E-BP1 such as T37 and T46 are priming sites and might not reflect the full activity of 4E-BP1 at the time-point of collection (Qin *et al*, 2016). In addition, S65 exhibits a higher degree of sensitivity to Rapamycin compared to other priming sites, serving as a better read-out for mTORC1 activity (Gingras *et al*, 2001).

This is now implemented in the revised version of the manuscript as:

Page 7: We checked p-4E-PB1^{Ser65} as other 4E-BP1 sites such as Thr37 and Thr46 are priming sites and might not fully reflect the activity of 4E-BP1 (Qin *et al*, 2016). In addition, p-4E-PB1^{Ser65} exhibits a higher degree of sensitivity to Rapamycin compared to other priming sites, serving as a better read-out for mTORC1 activity (Gingras *et al*, 2001).

10- Figs. 2i and Fig. 3a; the composite blots are clearly not all derived from the same gel/membrane. The authors should either show composites belonging to the same gel/membrane, or clearly label which composites correspond to the same gel/membrane.

We thank Referee #3 for these remarks. We now corrected the composite of the blots from Fig 2I and 3A in the main figures, specified those belonging to different membranes, and also provided the full composite of the blots in Appendix Figure S2.

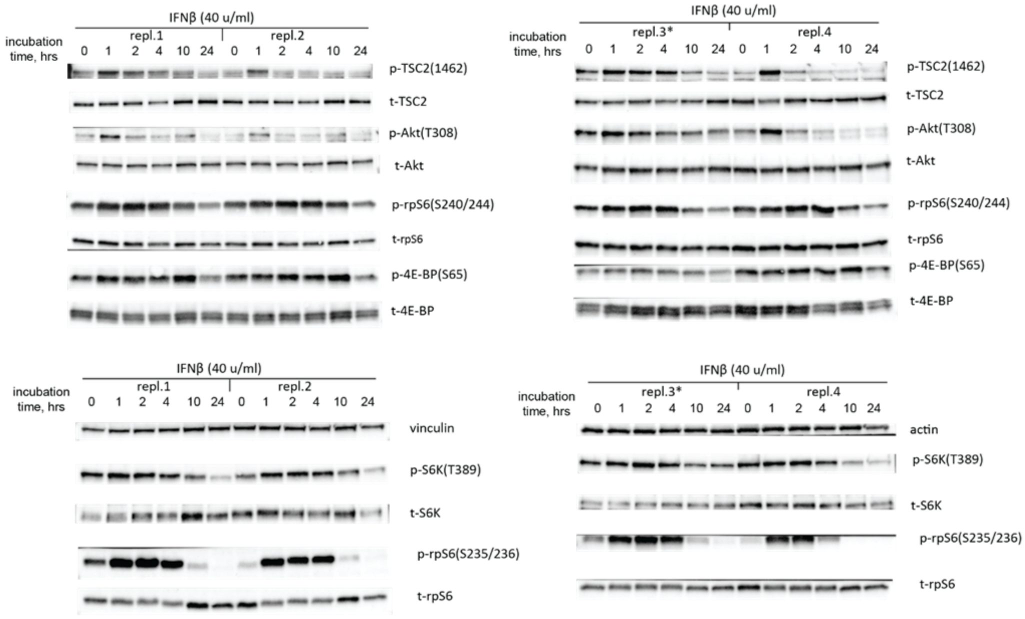

Appendix Figure S2. Collection of western blot membranes for assessing the biphasic response of mTOR caused by IFN-β treatment.

Collection of western blot membranes from 4 biological replicates of NSCs treated with IFN-β for the specified times. *Replicate 3 was used as representative blots for Figures 2I, 3A.

11- Figs. 3D and EV4F: Is there an increased in local 5'UTR footprint density in a subset of ISGs including Ifit3 and Irf9?

We now checked the local 5'UTR footprint density for the 300 ISGs upregulated upon IFN-β treatment in NSCs (NSC Type-I Interferon Response). However, out of 300 ISGs we could only find two genes (*Pttg1* and *Rbms2*) that were classified under the uORF group (Fig 3D), which we do not consider relevant for our manuscript. *Ifit3* and *Irf9* were also not classified as uORF genes.

This is now implemented in the revised version of the manuscript as:

Page 9: IFN-β increased local 5'UTR footprint density in a subset of genes while maintaining unaltered levels in the CDS, indicating alternative uORF usage (Fig 3D). Notably, ISGs were not among these transcripts with increased uORF usage.

12- Fig. S3a: Blots for WT and Mut tissue must be on the same membrane to ensure same transfer of protein and exposure time during acquisition.

This suggestion from Referee #3 might not be based on the submitted version of the manuscript.

We now repeated and updated the blot showing all controls and clones in the same blot in Fig EV4A:

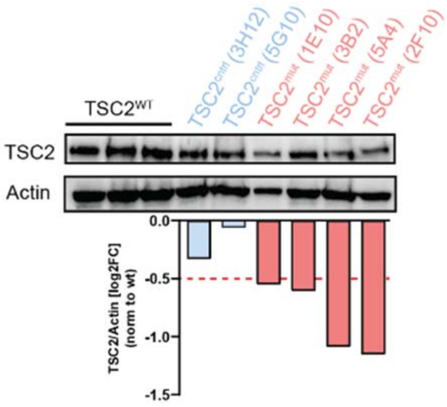

13- Figs. 3e and f: Can the authors provide direct evidence that TSC1/2 is being regulated by Cdk4/6 as proposed? Perhaps a western for TSC2 Ser1452 and Ser1217.

For clarification, the regulation of TSC1/2 by Cdk4/6 is already reported (Romero-Pozuelo *et al*, 2020). Our work describes that interferon modulates Cdk4/6 in NSCs and that such

modulation influences the activity of TSC2, as our scheme depicts (Fig 3B). As suggested by Referee #3, we now checked Cdk4/6-dependent phosphorylation at TSC2. We could not find a mouse-compatible anti-phospho $TSC2^{Ser1217}$ antibody, but we employed an anti-phospho $TSC2^{Ser1452}$ antibody kindly provided by the authors of Romero-Pozuelo *et al.* 2020. We find that this phosphorylation is reduced upon IFN-β treatment in NSCs, in line with the displayed inhibition of Cdk4/6.

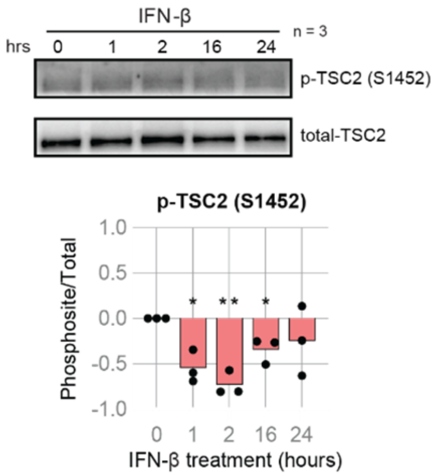

This is now implemented in Fig EV4G and the revised version of the manuscript as:

Page 9: Similarly, IFN-β also reduced the Cdk4/6-dependent phosphorylation at $TSC2^{Ser1452}$ (Romero-Pozuelo *et al*, 2020), consistent with the IFN-mediated inhibition of Cdk4/6 (Fig EV4G).

14- Authors need to show Sox2 protein levels to confirm that the transition of an activated stem cell into a differentiated neuroblast is mediated by Sox2 and controlled by IFN type I.

This suggestion from Referee #3 might not be based on the submitted version of the manuscript.

We thank Referee #3 for this experimental suggestion. We would like to emphasize that we do not claim that interferon promotes "the transition of an activated stem cell into a differentiated neuroblast". As discussed in the submitted version of the manuscript: "In this recent study, we addressed the role of repression of Sox2 translation in the transition of an activated stem cell into a differentiated neuroblast (Baser *et al*, 2019). Sox2 levels are however likewise reduced in quiescent NSCs to avoid replicative stress (Marqués-Torrejón *et al*, 2013). The determinants of directionality into a differentiated progenitor or a quiescent stem cell state following repression of Sox2 remain subject of future studies.".

We are glad to share with Referee #3 that we find a mild reduction in Sox2 protein in NSCs treated with IFN-β. Given that total protein levels reflect not only de-novo translation, but also degradation and protein stability, this validation supports the relevance of protein translation control in NSCs.

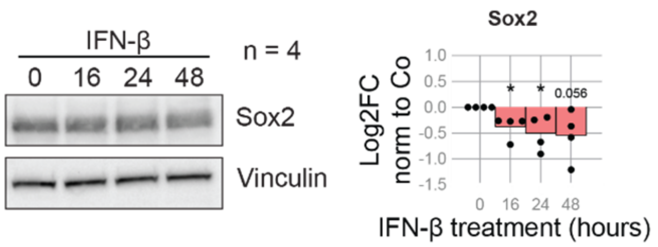

This is now implemented in Fig EV5A and the revised version of the manuscript as:

Page 10: This repression was also reflected in a mild reduction in Sox2 protein (Fig EV5A).

15- In the discussion, the wording of the following statements needs to be changed: "Our study shows that in the natural environment of the brain, the ISGs expressed upon exposure to Type I interferon in NSCs ex-vivo...". This is not correct since ex-vivo is not the same as the natural environment of the brain nor is the experimental manipulation of IFN-β levels.

This suggestion from Referee #3 might not be based on the submitted version of the manuscript.

We could not find the quoted sentence in the current manuscript.

The sentence that the authors refer to belongs to a previous version of the manuscript and was already changed to "Our study shows that in the natural environment of the brain, type-I interferon ISGs are highest expressed in NSCs but also present, albeit to a lower extent, in fully differentiated new-born neurons.".

References:

Almada AE, Horwitz N, Price FD, Gonzalez AE, Ko M, Bolukbasi OV, Messemer KA, Chen S, Sinha M, Rubin LL, *et al* (2021) FOS licenses early events in stem cell activation driving skeletal muscle regeneration. *Cell Rep* 34: 108656

Baruch K, Deczkowska A, David E, Castellano JM, Miller O, Kertser A, Berkutzki T, Barnett-Itzhaki Z, Bezalel D, Wyss-Coray T, *et al* (2014) Aging-induced type I interferon response at the choroid plexus negatively affects brain function. *Science (80-)* 346: 89 LP – 93

Baser A, Skabkin M, Kleber S, Dang Y, Gülcüler Balta GS, Kalamakis G, Göpferich M, Carvajal Ibañez D, Schefzik R, Lopez AS, *et al* (2019) Onset of differentiation is post-transcriptionally controlled in adult neural stem cells. *Nature* 566: 100–104

Cho IJ, Lui PPW, Obajdin J, Riccio F, Stroukov W, Willis TL, Spagnoli F & Watt FM (2019) Mechanisms, Hallmarks, and Implications of Stem Cell Quiescence. *Stem Cell Reports* 12: 1190–1200

Dulken BW, Buckley MT, Navarro Negredo P, Saligrama N, Cayrol R, Leeman DS, George BM, Boutet SC, Hebestreit K, Pluvinage J V., *et al* (2019) Single-cell analysis reveals T cell infiltration in old neurogenic niches. *Nature*

Gingras AC, Raught B & Sonenberg N (2001) Regulation of translation initiation by FRAP/mTOR. *Genes Dev* 15: 807–826

Heidt T, Sager HB, Courties G, Dutta P, Iwamoto Y, Zaltsman A, Von Zur Muhlen C, Bode C, Fricchione GL, Denninger J, *et al* (2014) Chronic variable stress activates hematopoietic stem cells. *Nat Med* 20: 754–758

Hsu PP, Kang SA, Rameseder J, Zhang Y, Ottina KA, Lim D, Peterson TR, Choi Y, Gray NS, Yaffe MB, et al (2011) The mTOR-regulated phosphoproteome reveals a mechanism of mTORC1-mediated inhibition of growth factor signaling. Science (80-) 332: 1317–1322

Jia JJ, Lahr RM, Solgaard MT, Moraes BJ, Pointet R, Yang AD, Celucci G, Graber TE, Hoang HD, Niklaus MR, *et al* (2021) MTORC1 promotes TOP mRNA translation through site-specific phosphorylation of LARP1. *Nucleic Acids Res* 49: 3461–3489

Kalamakis G, Brüne D, Ravichandran S, Bolz J, Ziebell F, Stiehl T, Catalá-Martinez F, Kupke J, Zhao S, Llorens-Bobadilla E, *et al* (2019) Quiescence Modulates Stem Cell Maintenance and Regenerative Capacity in the Aging Brain Article Quiescence Modulates Stem Cell Maintenance and Regenerative Capacity in the Aging Brain.

Llorens-Bobadilla E, Zhao S, Baser A, Saiz-Castro G, Zwadlo K & Martin-Villalba A (2015) Single-Cell Transcriptomics Reveals a Population of Dormant Neural Stem Cells that Become Activated upon Brain Injury. *Cell Stem Cell* 17: 329–340

Marqués-Torrejón MÁ, Porlan E, Banito A, Gómez-Ibarlucea E, Lopez-Contreras AJ, Fernández-Capetillo Ó, Vidal A, Gil J, Torres J & Fariñas I (2013) Cyclin-dependent kinase inhibitor p21 controls adult neural stem cell expansion by regulating Sox2 gene expression. *Cell Stem Cell* 12: 88–100

Qin X, Jiang B & Zhang Y (2016) 4E-BP1, a multifactor regulated multifunctional protein. *Cell Cycle* 15: 781–786

Romero-Pozuelo J, Figlia G, Kaya O, Martin-Villalba A & Teleman AA (2020) Cdk4 and Cdk6 Couple the Cell-Cycle Machinery to Cell Growth via mTORC1. *Cell Rep* 31

Sidrauski C, McGeachy AM, Ingolia NT & Walter P (2015) The small molecule ISRIB reverses the effects of eIF2α phosphorylation on translation and stress granule assembly. *Elife* 2015: 1–16

Thoreen CC, Chantranupong L, Keys HR, Wang T, Gray NS & Sabatini DM (2012) A unifying model for mTORC1-mediated regulation of mRNA translation. *Nature* 485: 109–113

Urbán N & Cheung TH (2021) Stem cell quiescence: The challenging path to activation. *Dev* 148

Wu X, Dao Thi VL, Huang Y, Billerbeck E, Saha D, Hoffmann HH, Wang Y, Silva LAV, Sarbanes S, Sun T, *et al* (2018) Intrinsic Immunity Shapes Viral Resistance of Stem Cells. *Cell* 172: 423-438.e25

14th Dec 2022

Dear Ana,

Thank you for the submission of your revised manuscript to EMBO Molecular Medicine. We have now received the enclosed report from the two referees who agreed to re-assess it. As you will see, the referees are now supportive, and I am pleased to inform you that we will be able to accept your manuscript pending the following amendments:

1. The remaining minor concerns of both referees.

On a more editorial level:

2. Please provide up to 5 keywords and incorporate them into the main text.

3. Remove the blue color font.

4. Figures and EV Tables should be removed from the main manuscript file.

5. Move The Paper Explained to the main manuscript file.

6. Remove the Author Contribution section from the manuscript text.

7. Figure callout is missing for Figure 4F; please fix it.

8. The Tables EV3-5 are rather large. Please rename them as Datasets EV1-3 and update the callouts accordingly.

9. We updated our journal's competing interests policy in January 2022 and request authors to consider both actual and perceived competing interests. Please review the policy https://www.embopress.org/competing-interests and update your competing interests if necessary.
Please use the heading "Disclosure statement and competing interests" and add "Ana Martin-Villalba is an EMBO member. This has no bearing on the editorial consideration of this article for publication."

10. Data availability: please make sure the GSE197217 will be made publicly available upon the acceptance of the manuscript.

11. Remove the Illustrations section.

12. Appendix: please rename the Figures and Tables in the "Appendix supplementary methods" section as "Appendix Figure Sx" and "Appendix Table Sx". Please update the callouts and table of content accordingly.

13. I have slightly modified and shortened the synopsis text (see attached). Please let me know if you would like to introduce further modifications.

14. Our data editors have seen the manuscript, and they have made some comments and suggestions that need to be addressed (see attached). Please send back a revised version (in track change mode), as we will need to go through the changes.

15. As part of the EMBO Publications transparent editorial process initiative (see our Editorial at http://embomolmed.embopress.org/content/2/9/329), EMBO Molecular Medicine will publish online a Review Process File (RPF) to accompany accepted manuscripts.

In the event of acceptance, this file will be published in conjunction with your paper and will include the anonymous referee reports, your point-by-point response, and all pertinent correspondence relating to the manuscript. Let us know whether you disagree with this and if you want to remove or keep any figures from it prior to publication.

Please note that the Authors checklist will be published at the end of the RPF.

Please submit your revised manuscript by December 20th and ideally as soon as possible.

I look forward to receiving a new revised version of your manuscript.

Kind regards,
Jingyi

Jingyi Hou
Editor
EMBO Molecular Medicine

***** Reviewer's comments *****

Referee #1 (Remarks for Author):

The authors have done a good job in the revision, fully answered my questions, and clarified my concerns. I support the acceptance of the manuscript for publication.

Some minor mistakes/missing:
Fig2F, labeled wrong, should be 14h.
Some new references are missing from the list of the main text, such as Qin 2016, Gingras 2001.

Referee #3 (Comments on Novelty/Model System for Author):

I have no further comments

Referee #3 (Remarks for Author):

The authors performed large numbers of new experiments and addressed all my comments. They insist on using the term "the exit of stem cell activation", which in my opinion is incorrect. The reference they provide does not clarify the meaning. Therefore, I encourage the authors to use "induce quiescence" instead of "the exit of stem cell activation", and only use "exit" for "exit from the quiescent state "or "mitotic exit". Likewise, "the ability of stem cells to activate. " should be replaced by "the ability of stem cells to become/get activated. English editing will improve the content quality.

Point-by-point responses to Review of EMM-2022-16434

Revision II (Pre-accepted pending the following amendments)

Referee's reports and point-by-point responses

Referee's comments are coloured in orange and changes are highlighted in blue.

Referee #1 (Remarks for Author):

The authors have done a good job in the revision, fully answered my questions, and clarified my concerns. I support the acceptance of the manuscript for publication.

We thank Referee #1 for its positive consideration.

Some minor mistakes/missing:

Fig2F, labeled wrong, should be 14h.

Thank you for noticing. We now changed the wrongly labelled axis of Figure 2F.

Some new references are missing from the list of the main text, such as Qin 2016, Gingras 2001.

We now included these missing references and revised the literature of the paper.

Referee #3 (Comments on Novelty/Model System for Author):

I have no further comments

Referee #3 (Remarks for Author):

The authors performed large numbers of new experiments and addressed all my comments.

We thank Referee #3 for its consideration and gratitude to the additional experiments and results.

They insist on using the term "the exit of stem cell activation", which in my opinion is incorrect. The reference they provide does not clarify the meaning. Therefore, I encourage the authors to use "induce quiescence" instead of "the exit of stem cell activation", and only use "exit" for "exit from the quiescent state "or "mitotic exit". Likewise, "the ability of stem cells to activate. " should be replaced by "the ability of stem cells to become/get activated. English editing will improve the content quality.

16th Dec 2022

Dear Ana,

I am pleased to inform you that your manuscript is accepted for publication and is now being sent to our publisher to be included in the next available issue of EMBO Molecular Medicine.

We would like to remind you that as part of the EMBO Publications transparent editorial process initiative, EMBO Molecular Medicine will publish a Review Process File online to accompany accepted manuscripts. If you do NOT want the file to be published or would like to exclude figures, please immediately inform the editorial office via e-mail.

Please read below for additional IMPORTANT information regarding your article, its publication and the production process.

Congratulations on this exciting work!

Jingyi

Jingyi Hou
Editor
EMBO Molecular Medicine

Follow us on Twitter @EmboMolMed
Sign up for eTOCs at embopress.org/alertsfeeds

*** *** *** IMPORTANT INFORMATION *** *** ***

SPEED OF PUBLICATION
The journal aims for rapid publication of papers, using using the advance online publication "Early View" to expedite the process: A properly copy-edited and formatted version will be published as "Early View" after the proofs have been corrected. Please help the Editors and publisher avoid delays by providing e-mail address(es), telephone and fax numbers at which author(s) can be contacted.

Should you be planning a Press Release on your article, please get in contact with embomolmed@wiley.com as early as possible, in order to coordinate publication and release dates.

LICENSE AND PAYMENT:

All articles published in EMBO Molecular Medicine are fully open access: immediately and freely available to read, download and share.

EMBO Molecular Medicine charges an article processing charge (APC) to cover the publication costs. You, as the corresponding author for this manuscript, should have already received a quote with the article processing fee separately. Please let us know in case this quote has not been received.

Once your article is at Wiley for editorial production you will receive an email from Wiley's Author Services system, which will ask you to log in and will present you with the publication license form for completion. Within the same system the publication fee can be paid by credit card, an invoice, pro forma invoice or purchase order can be requested.

Payment of the publication charge and the signed Open Access Agreement form must be received before the article can be published online.

PROOFS

You will receive the proofs by e-mail approximately 2 weeks after all relevant files have been sent o our Production Office. Please return them within 48 hours and if there should be any problems, please contact the production office at embopressproduction@wiley.com.

Please inform us if there is likely to be any difficulty in reaching you at the above address at that time. Failure to meet our deadlines may result in a delay of publication.

All further communications concerning your paper proofs should quote reference number EMM-2022-16434-V3 and be directed to the production office at embopressproduction@wiley.com.

Thank you,

Jingyi Hou
Editor
EMBO Molecular Medicine

EMBO Press Author Checklist

Corresponding Author Name: Ana Martin-Villalba, Anna Marciniak-Czochra	
Journal Submitted to: EMBO Molecular Medicine	
Manuscript Number: EMM-2022-16434	

USEFUL LINKS FOR COMPLETING THIS FORM
The EMBO Journal - Author Guidelines
EMBO Reports - Author Guidelines
Molecular Systems Biology - Author Guidelines
EMBO Molecular Medicine - Author Guidelines

Reporting Checklist for Life Science Articles (updated January 2022)
This checklist is adapted from Materials Design Analysis Reporting (MDAR) Checklist for Authors. MDAR establishes a minimum set of requirements in transparent reporting in the life sciences (see Statement of Task: 10.31222/osf.io/9sm4x). Please follow the journal's guidelines in preparing your manuscript.
Please note that a copy of this checklist will be published alongside your article.

Abridged guidelines for figures
1. Data
The data shown in figures should satisfy the following conditions:
- the data were obtained and processed according to the field's best practice and are presented to reflect the results of the experiments in an accurate and unbiased manner.
- ideally, figure panels should include only measurements that are directly comparable to each other and obtained with the same assay.
- plots include clearly labeled error bars for independent experiments and sample sizes. Unless justified, error bars should not be shown for technical replicates.
- if n<5, the individual data points from each experiment should be plotted. Any statistical test employed should be justified.
- Source Data should be included to report the data underlying figures according to the guidelines set out in the authorship guidelines on Data Presentation.

2. Captions
Each figure caption should contain the following information, for each panel where they are relevant:
- a specification of the experimental system investigated (eg cell line, species name).
- the assay(s) and method(s) used to carry out the reported observations and measurements.
- an explicit mention of the biological and chemical entity(ies) that are being measured.
- an explicit mention of the biological and chemical entity(ies) that are altered/varied/perturbed in a controlled manner.
- the exact sample size (n) for each experimental group/condition, given as a number, not a range;
- a description of the sample collection allowing the reader to understand whether the samples represent technical or biological replicates (including how many animals, litters, cultures, etc.).
- a statement of how many times the experiment shown was independently replicated in the laboratory.
- definitions of statistical methods and measures:
  - common tests, such as t-test (please specify whether paired vs. unpaired), simple χ2 tests, Wilcoxon and Mann-Whitney tests, can be unambiguously identified by name only, but more complex techniques should be described in the methods section;
  - are tests one-sided or two-sided?
  - are there adjustments for multiple comparisons?
  - exact statistical test results, e.g., P values = x but not P values < x;
  - definition of 'center values' as median or average;
  - definition of error bars as s.d. or s.e.m.

Please complete ALL of the questions below.
Select "Not Applicable" only when the requested information is not relevant for your study.

Materials

Newly Created Materials	Information included in the manuscript?	In which section is the information available? (Reagents and Tools Table, Materials and Methods, Figures, Data Availability Section)
New materials and reagents need to be available; do any restrictions apply?	Yes	Data availability

Antibodies	Information included in the manuscript?	In which section is the information available? (Reagents and Tools Table, Materials and Methods, Figures, Data Availability Section)
For **antibodies** provide the following information:<br>- Commercial antibodies: RRID (if possible) or supplier name, catalogue number and or/clone number<br>- Non-commercial: RRID or citation	Yes	Materials and Methods

DNA and RNA sequences	Information included in the manuscript?	In which section is the information available? (Reagents and Tools Table, Materials and Methods, Figures, Data Availability Section)
**Short novel DNA or RNA including primers, probes**: provide the sequences.	Yes	Materials and Methods, Expanded View tables

Cell materials	Information included in the manuscript?	In which section is the information available? (Reagents and Tools Table, Materials and Methods, Figures, Data Availability Section)
**Cell lines:** Provide species information, strain. Provide accession number in repository **OR** supplier name, catalog number, clone number, and/**OR** RRID.	Not Applicable	
**Primary cultures:** Provide species, strain, sex of origin, genetic modification status.	Yes	Materials and Methods
Report if the cell lines were recently **authenticated** (e.g., by STR profiling) and tested for mycoplasma contamination.	Not Applicable	

Experimental animals	Information included in the manuscript?	In which section is the information available? (Reagents and Tools Table, Materials and Methods, Figures, Data Availability Section)
**Laboratory animals or Model organisms:** Provide species, strain, sex, age, genetic modification status. Provide accession number in repository **OR** supplier name, catalog number, clone number, **OR** RRID.	Yes	Materials and Methods
**Animal observed in or captured from the field:** Provide species, sex, and age where possible.	Not Applicable	
Please detail **housing and husbandry conditions**.	Yes	Materials and Methods

Plants and microbes	Information included in the manuscript?	In which section is the information available? (Reagents and Tools Table, Materials and Methods, Figures, Data Availability Section)
**Plants:** provide species and strain, ecotype and cultivar where relevant, unique accession number if available, and source (including location for collected wild specimens).	Not Applicable	
**Microbes:** provide species and strain, unique accession number if available, and source.	Not Applicable	

Human research participants	Information included in the manuscript?	In which section is the information available? (Reagents and Tools Table, Materials and Methods, Figures, Data Availability Section)
If collected and within the bounds of privacy constraints report on age, sex and gender or ethnicity for all study participants.	Not Applicable	

Core facilities	Information included in the manuscript?	In which section is the information available? (Reagents and Tools Table, Materials and Methods, Figures, Data Availability Section)
If your work benefited from core facilities, was their service mentioned in the acknowledgments section?	Yes	Acknowledgments

Design

Study protocol	Information included in the manuscript?	In which section is the information available? (Reagents and Tools Table, Materials and Methods, Figures, Data Availability Section)
If study protocol has been **pre-registered, provide DOI in the manuscript**. For clinical trials, provide the trial registration number **OR** cite DOI.	Not Applicable	
Report the **clinical trial registration number** (at ClinicalTrials.gov or equivalent), where applicable.	Not Applicable	

Laboratory protocol	Information included in the manuscript?	In which section is the information available? (Reagents and Tools Table, Materials and Methods, Figures, Data Availability Section)
Provide DOI OR other citation details if **external detailed step-by-step protocols** are available.	Not Applicable	

Experimental study design and statistics	Information included in the manuscript?	In which section is the information available? (Reagents and Tools Table, Materials and Methods, Figures, Data Availability Section)
Include a statement about **sample size** estimate even if no statistical methods were used.	Not Applicable	
Were any steps taken to minimize the effects of subjective bias when allocating animals/samples to treatment (e.g. **randomization procedure**)? If yes, have they been described?	Not Applicable	
Include a statement about **blinding** even if no blinding was done.	Not Applicable	
Describe **inclusion/exclusion criteria** if samples or animals were excluded from the analysis. Were the criteria pre-established?<br><br>If sample or data points were omitted from analysis, report if this was due to attrition or intentional exclusion and provide justification.	Yes	Materials and Methods, Figure captions
For every figure, are **statistical tests** justified as appropriate? Do the data meet the assumptions of the tests (e.g., normal distribution)? Describe any methods used to assess it. Is there an estimate of variation within each group of data? Is the variance similar between the groups that are being statistically compared?	Yes	Materials and Methods, Figure captions

Sample definition and in-laboratory replication	Information included in the manuscript?	In which section is the information available? (Reagents and Tools Table, Materials and Methods, Figures, Data Availability Section)
In the figure legends: state number of times the experiment was **replicated** in laboratory.	Yes	Figure captions
In the figure legends: define whether data describe **technical or biological replicates**.	Yes	Figure captions

Ethics

Ethics	Information included in the manuscript?	In which section is the information available? (Reagents and Tools Table, Materials and Methods, Figures, Data Availability Section)
Studies involving **human participants**: State details of **authority granting ethics approval** (IRB or equivalent committee(s), provide reference number for approval.	Not Applicable	
Studies involving **human participants**: Include a statement confirming that **informed consent** was obtained from all subjects and that the experiments conformed to the principles set out in the WMA Declaration of Helsinki and the Department of Health and Human Services Belmont Report.	Not Applicable	
Studies involving **human participants:** For publication of **patient photos**, include a statement confirming that consent to publish was obtained.	Not Applicable	
Studies involving experimental **animals**: State details of **authority granting ethics approval** (IRB or equivalent committee(s), provide reference number for approval. Include a statement of compliance with ethical regulations.	Yes	Materials and Methods
Studies involving **specimen and field samples:** State if relevant **permits** obtained, provide details of authority approving study; if none were required, explain why.	Not Applicable	

Dual Use Research of Concern (DURC)	Information included in the manuscript?	In which section is the information available? (Reagents and Tools Table, Materials and Methods, Figures, Data Availability Section)
Could your study fall under dual use research restrictions? Please check biosecurity documents and list of **select agents and toxins** (CDC): https://www.selectagents.gov/sat/list.htm	Not Applicable	
If you used a select agent, is the security level of the lab appropriate and reported in the manuscript?	Not Applicable	
If a study is subject to dual use research of concern regulations, is the name of the **authority granting approval and reference number** for the regulatory approval provided in the manuscript?	Not Applicable	

Reporting

The MDAR framework recommends adoption of discipline-specific guidelines, established and endorsed through community initiatives. Journals have their own policy about requiring specific guidelines and recommendations to complement MDAR.

Adherence to community standards	Information included in the manuscript?	In which section is the information available? (Reagents and Tools Table, Materials and Methods, Figures, Data Availability Section)
State if relevant guidelines or checklists (e.g., **ICMJE, MIBBI, ARRIVE, PRISMA**) have been followed or provided.	Not Applicable	
For **tumor marker prognostic studies,** we recommend that you follow the **REMARK** reporting guidelines (see link list at top right). See author guidelines, under 'Reporting Guidelines'. Please confirm you have followed these guidelines.	Not Applicable	
For **phase II and III randomized controlled trials**, please refer to the **CONSORT** flow diagram (see link list at top right) and submit the CONSORT checklist (see link list at top right) with your submission. See author guidelines, under 'Reporting Guidelines'. Please confirm you have submitted this list.	Not Applicable	

Data Availability

Data availability	Information included in the manuscript?	In which section is the information available? (Reagents and Tools Table, Materials and Methods, Figures, Data Availability Section)
Have **primary datasets** been deposited according to the journal's guidelines (see 'Data Deposition' section) and the respective accession numbers provided in the Data Availability Section?	Yes	Materials and Methods; Data Availability Section
Were **human clinical and genomic datasets** deposited in a public access-controlled repository in accordance to ethical obligations to the patients and to the applicable consent agreement?	Not Applicable	
Are **computational models** that are central and integral to a study available without restrictions in a machine-readable form? Were the relevant accession numbers or links provided?	Yes	Materials and Methods; Data Availability Section
If publicly available data were reused, provide the respective **data citations in the reference list**.	Not Applicable	
